# ATP induces folding of ALS-causing C71G-hPFN1 and nascent hSOD1

**DOI:** 10.1038/s42004-023-00997-0

**Published:** 2023-09-05

**Authors:** Jian Kang, Liangzhong Lim, Jianxing Song

**Affiliations:** Department of Biological Sciences, Faculty of Science, National University of Singapore, 10 Kent Ridge Crescent, Singapore, 119260 Singapore

**Keywords:** Protein aggregation, Solution-state NMR, Biophysical chemistry, Enzyme mechanisms

## Abstract

ALS-causing C71G-hPFN1 coexists in both folded and unfolded states, while nascent hSOD1 is unfolded. So far, the mechanisms underlying their ALS-triggering potential remain enigmatic. Here we show by NMR that ATP completely converts C71G-hPFN1 into the folded state at a 1:2 ratio, while inducing nascent hSOD1 into two co-existing states at a 1:8 ratio. Surprisingly, the inducing capacity of ATP comes from its triphosphate, but free triphosphate triggers aggregation. The inducing capacity ranks as: ATP = ATPP = PPP > ADP = AMP−PNP = AMP−PCP = PP, while AMP, adenosine, P, and NaCl show no conversion. Mechanistically, ATP and triphosphate appear to enhance the intrinsic folding capacity encoded in the sequences, as unveiled by comparing conformations and dynamics of ATP- and Zn^2+^-induced hSOD1 folded states. Our study provides a mechanism for the finding that some single-cell organisms employ polyphosphates as primordial chaperones, and sheds light on the enigma of age-related onset of familial ALS and risk increase of neurodegenerative diseases.

## Abstract (web summary)

Amyotrophic lateral sclerosis (ALS) is associated with the misfolding of hSOD1 and C71G-hPFN1, however, the exact mechanism responsible for ALS remains poorly understood. Here, NMR spectroscopy reveals that ATP can induce the folding of hSOD1 and C71G-hPFN1, inhibiting protein aggregation and enhancing their thermodynamic stability.

## Introduction

**Fig. 1:**
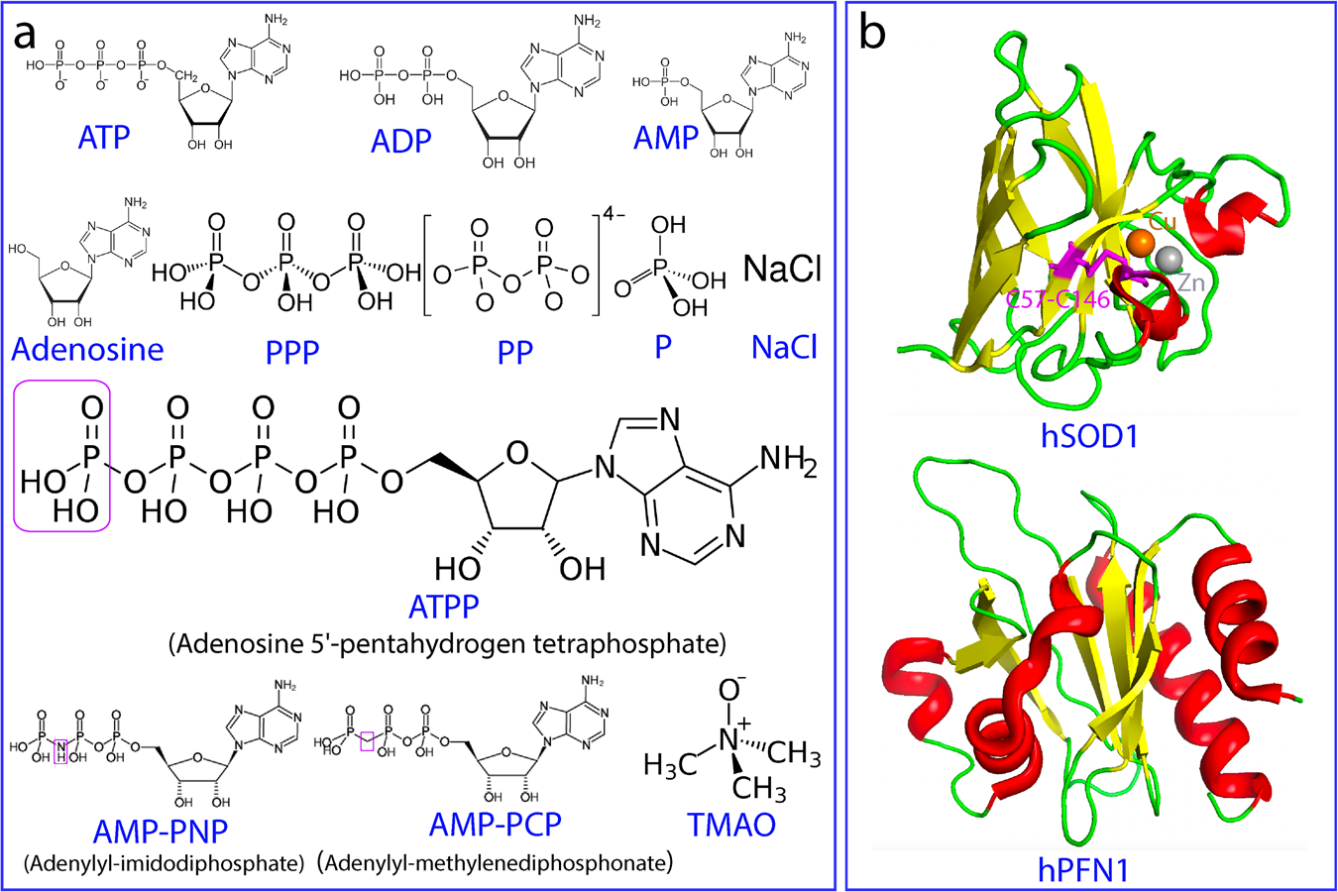
12 Small molecules and 2 proteins in the present study. **a** Chemical structures of ATP and 11 related compounds. **b** Three-dimensional structures of hSOD1 and hPFN1.

Many proteins need to fold from the unfolded state (U) into the folded state (F) for their functions^1–7^. On the other hand, the cell is extremely crowded, and protein concentrations can exceed 100 mg/ml^8^. As the folded state of some proteins is only marginally stable, genetic mutations are sufficient to destabilize them, thus leading to their misfolding/aggregation in cells, which is a common pathological hallmark of aging and neurodegenerative diseases^9–11^. Currently, it is widely thought that modern cells handle protein folding and misfolding/aggregation problems mainly with supramolecular machinery energetically driven by ATP (Fig. 1a), the universal energy currency that only requires micromolar concentrations for its previously-known functions^12,13^. Mysteriously, however, ATP has very high concentrations in all living cells ranging from 2 to 12 mM depending on cell type. For example, the vertebrate lens, a metabolically quiescent organ, still maintains ATP concentrations of >7 mM^13–17^.

Only recently, ATP has been decoded to energy-independently control protein hemostasis at concentrations >mM. In this context, ATP can behave as a biological hydrotrope to dissolve protein liquid-liquid phase separation (LLPS) and aggregates^15,16^. We also found that ATP can act as a bivalent binder to induce and subsequently dissolve LLPS of intrinsically-disordered proteins^17–20^ as well as to kinetically or/and thermodynamically inhibit amyloid fibrillation of well-folded domains^21,22^. In particular, ATP appears to also act as a hydration mediator to antagonize the crowding-induced destabilization of the human eye-lens protein γS-crystallin without specific binding^23^. Nevertheless, so far it remains unknown whether ATP can directly modulate protein folding equilibrium, the core event of protein hemostasis.

Amyotrophic lateral sclerosis (ALS) is the most common motor neuron disease which was first described in 1869 but its mechanism still remains a mystery^9–11^. Most ALS cases are sporadic (90%) (SALS) whereas 10% are familial ALS (FALS). In 1993, human CuZn-superoxide dismutase 1 (hSOD1) was identified to be the first causative gene whose mutations cause the most prevalent form of FALS^24–30^. Intriguingly, misfolding/aggregation of the wild-type (WT) hSOD1 has also been extensively found to be associated with SALS. So far, despite exhaustive studies, the exact mechanism for ALS still remains an enigma^24–30^.

hSOD1 is one of the most studied proteins and its mature form is a homodimeric enzyme of remarkably high stability and solubility, with each subunit folding into an eight-stranded Greek-key β-barrel stabilized by an intramolecular disulfide bridge Cys57-Cys146 and holding one copper and one zinc ions (Fig. 1b). In contrast, by NMR nascent hSOD1 was characterized to be completely unfolded^31,32^. In-cell studies revealed that unfolded nascent hSOD1 folds into the mature form through a very complex multi-step maturation process in which the critical first step is the initial folding specifically induced by Zn^2+^^33^. Subsequently, with the catalysis by a copper chaperone for hSOD1 (CCS), the disulfide bridge is formed and copper is incorporated^34–36^. As the early folding species have a very high tendency of misfolding/aggregation^24–37^, various super-stable hSOD1 mutants with Cys residues differentially mutated to Ala or/and Ser such as C6A/C111S mutants have been widely used for biophysical and NMR studies^34^.

140-residue human profilin 1 (hPFN1) physiologically regulates actin polymerisation^38^, which adopts a seven-stranded antiparallel β-sheet sandwiched by N- and C-terminal α-helices on one face of the sheet; and three small helical regions on the opposite face (Fig. 1b). Several mutations of hPFN1 have been identified to cause FALS, out of which C71G with Cys71 mutated to Gly is the most toxic and prone to misfolding/aggregation and consequently all attempts to determine its crystal structure has failed^39–41^. Strikingly, C71G-hPFN1 has been demonstrated to cause ALS phenotypes in mice by gain of toxicity but with unknown mechanism^42^. Previously, C71G-hPFN1 was shown by NMR to coexist between the unfolded and folded states characteristic of two sets of HSQC peaks, suggesting the existence of an energy barrier to separate its two states^43,44^.

To shed light on the mystery of ALS caused by hSOD1 and C71G-hPFN1, in the present study, we first aimed to map out NMR dynamics of the conformational equilibrium on both ps-ns and μs-ms time scales, as well as thermodynamic stability of C71G-hPFN1 and nascent hSOD1. Subsequently, nascent hSOD1 and C71G-hPFN1 were established as complementary models for directly visualizing the effect of 12 cations as well as ATP and 11 small molecules (Fig. 1a) on their folding and aggregation by NMR. The results provide the biophysical mechanisms for their high susceptibility to misfolding/aggregation. Most strikingly, ATP has been discovered to completely convert C71G-hPFN1 into the folded population even at a ratio of 1:2 (C71G:ATP) and to transform the nascent hSOD1 into an equilibrium of the folded and unfolded states at 1:8. By contrast, TMAO, the best-known molecule with the general capacity in inducing protein folding, failed to show any detectable induction even at ratios up to 1:2000 for C71G-hPFN1 and 1:1000 for hSOD1. Unexpectedly, the inducing capacity of ATP has been decoded to come from its triphosphate. However, although the isolated triphosphate (PPP) was able to induce the folding as effectively as ATP, it showed a strong ability to trigger aggregation. Results together imply that only by joining adenosine with triphosphate, nature marvelously creates ATP which owns three integrated abilities: effectively inducing protein folding, inhibiting aggregation, and enhancing thermodynamic stability. Our results provide a mechanism for the finding that inorganic polyphosphates can function as primordial chaperones^45,46^. Furthermore, ATP appears to continue to play a previously-unknown role in modern cells to energy-independently prevent protein misfolding/aggregation by enhancing the intrinsic folding capacity, thus rationalizing the long-standing puzzle that the individuals carrying ALS-causing mutants have FALS onset only after a certain age.

## Results

### NMR quantification of the conformational equilibrium of C71G-hPFN1

Here we first achieved sequential assignments of WT-hPFN1 and C71G-hPFN1 by collecting and analyzing a series of triple-resonance NMR spectra. As indicated by Supplementary Fig. 1a, WT-hPFN1 and the folded state of C71G-hPFN1 have highly similar chemical shift differences between Cα and Cβ (ΔCα-ΔCβ), an indicator for the secondary structure propensity^46^. This observation indicates that they have very similar secondary structures. By contrast, the absolute values of (ΔCα-ΔCβ) of the unfolded state are much smaller than those of its folded state (Supplementary Fig. 1b), clearly suggesting that the unfolded state has no stable secondary structures.

**Fig. 2:**
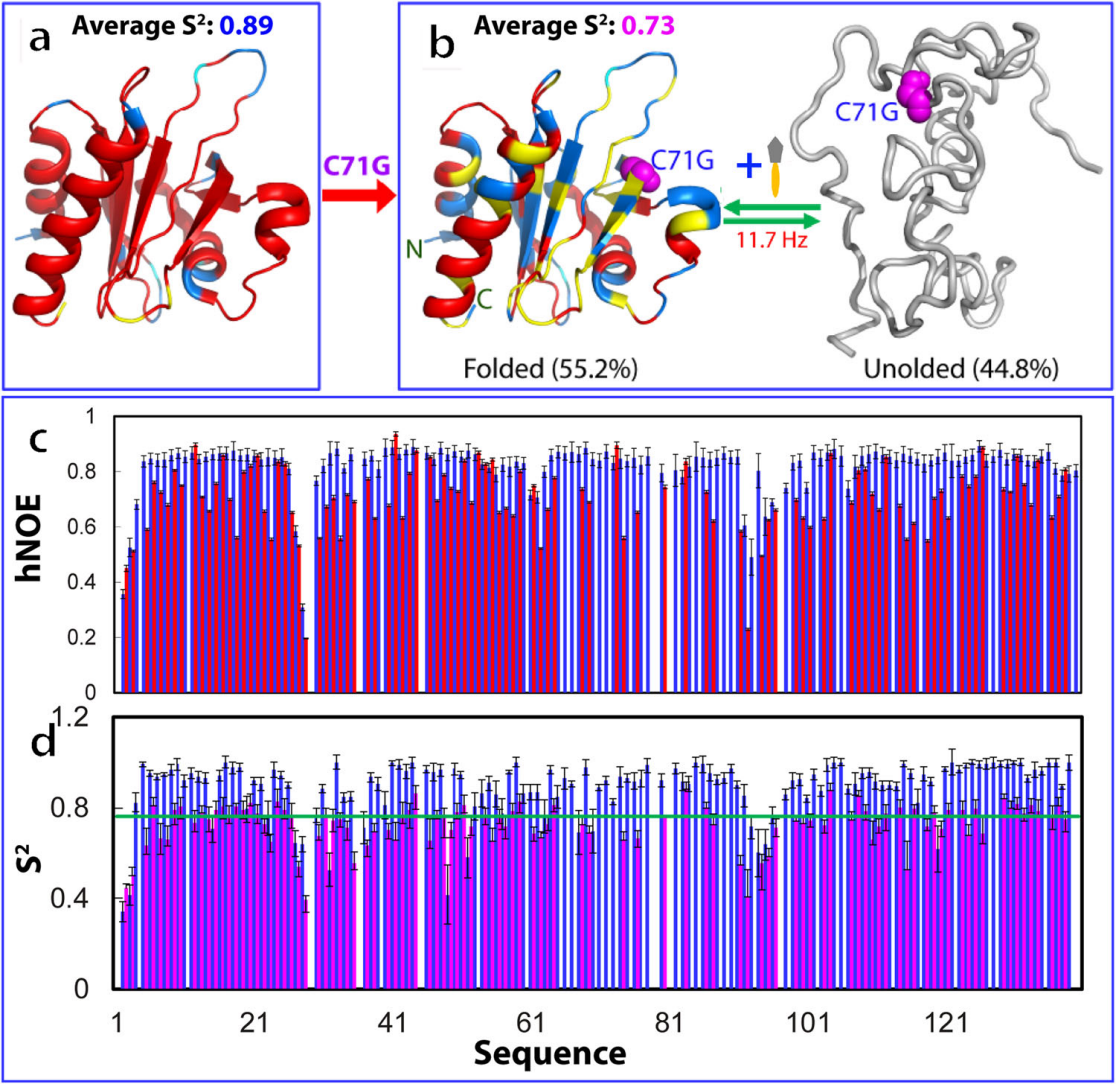
NMR quantification of ps-ns and μs-ms dynamics of the folding equilibrium. **a** The structure of WT-hPFN1 (PDB ID of 2PAV) with S^2^ values of WT-hPFN1 (the average value of 0.89) mapped on. **b** A diagram to show the co-existence of the folded (the average S^2^ value of 0.73) and unfolded states of C71G-hPFN1. The populations of the folded and unfolded states have been calculated to be respectively 55.2% and 44.8% which undergo a conformational exchange at 11.7 Hz. **c** {^1^H}-^15^N steady-state NOE intensities (hNOE) of WT-hPFN1 (blue) and the folded state of C71G-hPFN1 (red). **d** Generalized squared order parameter (S^2^) (II) of WT-hPFN1 (blue) and the folded state of C71G-hPFN1 (purple). The green line has a S^2^ value of 0.76 (average – STD of WT-hPFN1). S^2^ values are also mapped onto the folded state of C71G-hPFN1 in (**b**). Cyan is used for indicating Pro residues, and yellow for residues with missing or overlapping HSQC peaks. Red is for residues with S^2^ values > 0.76 and blue for residues with S^2^ values < 0.76. The error bars were generated during the fitting processes.

To characterize ps-ns backbone dynamics of WT-hPFN1 and C71G-hPFN1, here we collected their ^15^N backbone relaxation data R1, R2, and hNOE (Supplementary Fig. 2). Most residues of WT-hPFN1 have very large hNOE, with the average of 0.82 (Supplementary Fig. 2a), typical of a well-folded protein^46–50^. Subsequently, we performed the “model-free” analysis, which generates squared generalized order parameters, S^2^, reflecting the conformational rigidity on ps-ns time scale. S^2^ values range from 0 for a high internal motion to 1 for completely restricted motion in a molecular reference frame^47–50^. As shown in Fig. 2a, the majority of the WT-hPFN1 residues have S^2^ > 0.76 (with an average value of 0.89), suggesting that WT-hPFN1 has very high conformational rigidity.

We also decided to quantify the populations and exchange parameters for the folding equilibrium of C71G-hPFN1 by collecting and analyzing 3D heteronuclear HSQC-NOESY spectrum, which is a powerful version of ZZ-exchange experiments for resolving severe overlaps of NMR peaks for large proteins such as C71G-hPFN1 (Supplementary Fig. 3). However, even with 3D HSQC-NOESY, we could only successfully identify 8 residues (Supplementary Table 1) with well-resolved NOE peaks from the two states (folded/unfolded) and their exchange processes for accurately measuring their intensity. Fortunately, these 8 residues are spread across the entire sequence, thus providing insights into the global view of exchange dynamics. Their populations and exchange rates were successfully derived as described in Method and presented in Supplementary Table 1. The average populations of 8 residues are 55.2% and 44.8% respectively for the folded and unfolded states with an average exchange rate of ~11.7 Hz (~85.5 milli-second) (Supplementary Table 1).

We analyzed the relaxation data for C71G-hPFN1 (Supplementary Fig. 2). As shown in Fig. 2c, the folded state of C71G-hPFN1 has an average hNOE value of 0.70 with most residues having hNOE values smaller than those of WT-hPFN1, indicating that the folded state of C71G-hPFN1 has backbone dynamics higher than those of WT-hPFN1 on ps-ns time scale. To facilitate the comparison, we also performed the “model-free” analysis. As shown in Fig. 2d, many residues of the folded state of C71G-hPFN1 have S^2^ < 0.76 (Fig. 2b) (with an average value of 0.73), revealing that even the folded state of C71G-hPFN1 becomes more flexible than WT-hPFN1 on ps-ns time scale.

Furthermore, the overall rotational correlation times (τc) were calculated to be 7.5 ns and 7.8 ns respectively for WT-hPFN1 and C71G-hPFN1. The results imply that C71G-hPFN1 becomes slightly less compact. To independently confirm this, here by use of pulsed field gradient NMR self-diffusion measurements^51^, we measured their translational diffusion coefficients to be 1.12 ± 0.03 × 10^−10^ m^2^/s for WT-hPFN1, and 1.03 ± 0.02 × 10^−10^ m^2^/s for C71G-hPFN1, confirming that the folded state of C71G-hPFN1 indeed becomes less compact than WT-hPFN1.

### ATP induces folding of the unfolded state of C71G-hPFN1

Here, we first assessed whether ATP has any specific binding pocket on WT-hPFN1 by titrating ATP into the WT-PFN1 sample. As shown in I of Supplementary Fig. 4, even with the molar ratio up to 1:400 (hPFN1:ATP), ATP induced no large shift of HSQC peaks of WT-PFN1 except for those of His120 and Gly121. As His120 and Gly121 are located on an exposed loop (II of Supplementary Fig. 4), it is most likely that the shift is resulting from non-specific electrostatic effect from the highly negatively-charged ATP. The result suggests that unlike the folded nucleic-acid-binding domains^17^ such as FUS RRM^21^ with specific ATP-binding pockets, WT-hPFN1 without any known activity in binding nucleic acid also has no specific binding pocket for ATP.

**Fig. 3:**
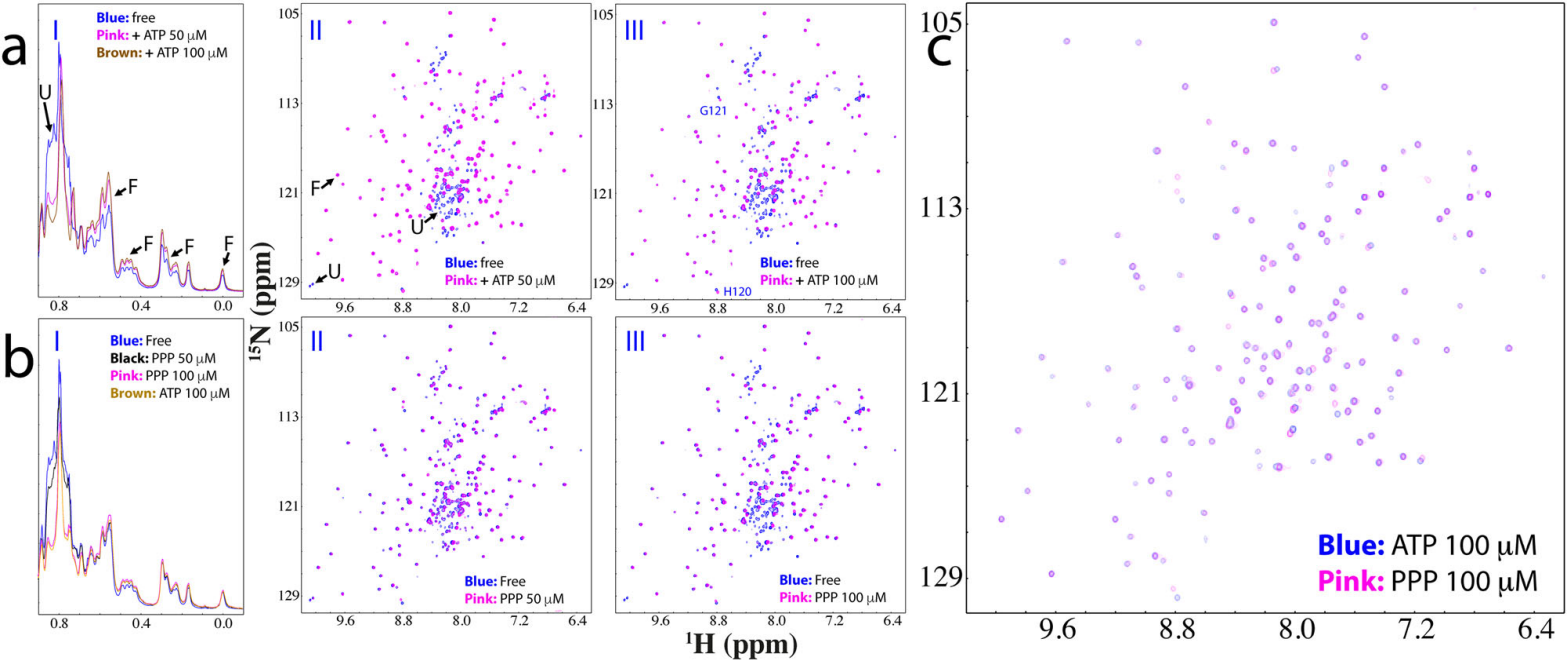
ATP or triphosphate completely converted the unfolded state into the folded state. **a** Up-field 1D NMR spectra and superimposition of HSQC spectra of ^15^N-labeled C71G-hPFN1 at a concentration of 50 μM in the absence (blue) or in the presence of ATP (pink) at different molar ratios. Some characteristic NMR signals of the folded (F) and unfolded (U) states were indicated by arrows. Two residues (His120 and Gly121) of the folded state with shifted HSQC peaks are labeled. **b** Up-field 1D NMR spectra and superimposition of HSQC spectra of C71G-hPFN1 at a concentration of 50 μM in the absence (blue) or in the presence of Triphosphate (pink) at different molar ratios. **c** Superimposition of HSQC spectra of C71G-hPFN1 in the presence of ATP at 1:2 (blue) and PPP (pink) at 1:2.

Strikingly, when ATP was added into the C71G-hPFN1 sample even only at a molar ratio of 1:0.5 (C71G:ATP), the intensity of HSQC peaks of the unfolded state became reduced while those of the folded state slightly increased (Supplementary Fig. 5 and S6). Briefly, the 1D peak intensity of the methyl group from the unfolded state also became reduced while those of the folded state slightly increased (Supplementary Fig. 5). When ATP was added to 1:1, the HSQC peak intensity of the unfolded state became further reduced while those of the folded state increased (I of Fig. 3a and S5). At 1:2, the 1D peak intensity of the methyl group of the folded state further increased (I of Fig. 3a) while HSQC peaks of the unfolded state completely disappeared (III of Fig. 3a). Further addition of ATP to 1:20 (1 mM) only induced slight shifts of some HSQC peaks of the folded state (IV of Fig S5). These results indicate that even at 1:2, ATP is capable of completely shifting the conformational equilibrium to the folded state. Interestingly, no considerable shift was observed for HSQC peaks of the folded state of C71G-hPFN1 except for those of His120 and Gly121 (III of Fig. 3a). This clearly indicates that like WT-hPFN1 (Supplementary Fig. 4), C71G-hPFN1 also has no specific binding pocket for ATP. We also increased the C71G-hPFN1 concentration to 100 μM, and the ATP concentration required to completely convert the unfolded state also doubled (200 μM). This result suggests that the conversion by ATP is dependent of the molar ratio between C71G-hPFN1 and ATP.

### ADP, AMP, and adenosine have differential inducing capacity

To determine the group and mechanism of ATP molecule responsible for the inducing capacity, we systematically titrated C71G-hPFN1 with a list of 10 related molecules (Fig. 1). For ADP, only at 1:8 (C71G:ADP), HSQC peaks of the unfolded state became completely converted into the folded state. This indicates that ADP still has the capacity to induce folding of the unfolded state but the capacity is much weaker than that of ATP. Strikingly, HSQC peaks with ATP at 1:8 are highly superimposable to those with ADP at 1:40 (Supplementary Fig. 7d), indicating that ADP also has no strong binding. We also titrated with AMP (Supplementary Fig. 8), but even with concentrations up to 20 mM (1:400), AMP was still unable to completely convert the unfolded state into the folded state (Supplementary Fig. 8d). We further titrated with adenosine and even with the highest concentrations of 5 mM due to its low solubility, no significant change was detected (Supplementary Fig. 9). The results strongly imply that the capacity of ATP in inducing the folding likely comes from its triphosphate group.

### The inducing capacity of ATP comes from its triphosphate group

We then systematically titrated C71G-hPFN1 with sodium salts of triphosphate (PPP), pyrophosphate (PP), and phosphate (P). For PPP, at 1:1 (C71G:PPP), the HSQC peak intensity of the unfolded state became significantly reduced (Fig. 3b and Supplementary Fig. 10). Furthermore, the 1D peak intensity of the methyl group of the unfolded state also became reduced while those of the folded state slightly increased (Fig. 3b). At 1:2, HSQC peaks of the unfolded state became completely disappeared (III of Fig. 3b), and the intensity of the methyl group of the folded state further increased (I of Fig. 3b). Very strikingly, C71G-hPFN1 with PPP at 1:2 has both HSQC (Fig. 3c) and 1D (I of Fig. 3b) spectra very similar to those with ATP at 1:2. The result suggests that ATP and PPP induce the folding of C71G-hPFN1 with the highly-similar effectiveness and mechanism.

On the other hand, the addition of PPP at 1:4 resulted in the broadening of NMR signals, and after one hour the sample formed white precipitates, and NMR signals became too weak to be detectable. The results revealed that although the capacity of ATP in inducing the folding comes from its triphosphate group, the free triphosphate owns a very strong ability to induce severe aggregation of C71G-hPFN1. Mechanistically, as we previously showed^11^, highly disordered and partially-folded proteins have many exposed hydrophobic patches Consequently, they will be driven to aggregate by hydrophobic interactions upon being exposed to charged molecules which can impose electrostatic screening effects^11^. Here as revealed by the above NMR studies on both ps-ns and μs-ms dynamics, not only the unfolded state of C71G-hPFN1 is highly disordered, its folded state also has an increased ns-ps dynamics. As such, even though the unfolded state of C71G-hPFN1 was completely converted into the folded state by PPP, the folded state of C71G-hPFN1 still has mutation-causing packing defects and dynamically exposed hydrophobic patches, which will be unavoidably triggered to misfold/aggregate in the presence of the highly negatively charged PPP. By contrast, the aromatic base ring of ATP might dynamically interact with the exposed hydrophobic patches of C71G-hPFN1 to shield the screening effects imposed by the triphosphate group to some extent, thus attenuating the aggregation induced by PPP.

Indeed, we further titrated with pyrophosphate (PP). As shown in Supplementary Fig. 11, at 1:6 (C71G:PP), the HSQC peaks of the unfolded state are still retained despite the reduction of their intensity. At 1:8, although the HSQC peaks of the unfolded state disappeared, the intensity of 1D and HSQC peaks of the folded state also reduced. In particular, after about one hour, the visible precipitates could be observed and NMR signals become too weak to be detectable. Subsequently, we carried out titrations with sodium phosphate (Supplementary Fig. 12) and chloride (Supplementary Fig. 13), and both failed to convert the unfolded state into the folded state with a concentration of up to 5 mM (1:100) for sodium phosphate (Supplementary Fig. 12), and 10 mM (1:200) for NaCl (Supplementary Fig. 13), where NMR samples started to show visible precipitation.

These results with PPP, PP, P, and NaCl strongly imply that the inducing capacity of PPP and PP is not due to the screening effect because the ionic strength of sodium triphosphate (PPP) and sodium pyrophosphate (PP) are maximally 15-times and 10-times stronger than sodium chloride while only 2.5-times and ~1.7-times stronger than sodium phosphate^52^. Nevertheless, sodium chloride at 10 mM with an ionic strength much larger than those of PPP at 0.1 mM and PP at 0.4 mM showed no capacity at all inducing the folding of the unfolded state but trigger the aggregation of C71G-hPFN1.

### The inducing capacity depends on the atoms linking the beta and gamma phosphates

We further assessed the effects on the folding equilibrium of three nonnatural ATP analogs, namely Adenosine 5’-(pentahydrogen tetraphosphate) (ATPP), Adenylyl-imidodiphosphate (AMP-PNP), and Methyleneadenosine 5-triphosphate (AMP-PCP) (Fig. 1). Very unexpectedly, ATPP induced the complete conversion of the unfolded state into the folded state at 1:2 (C71G:ATPP) which is very similar to ATP (Supplementary Fig. 14a). This result indicates that the inclusion of an extra phosphate failed to increase the capacity of ATP to induce folding. By contrast, ATPP showed a much higher ability than ATP to trigger the aggregation of the C71G-hPFN1 protein as evidenced by the result at 1:4 (C71G:ATPP), the C71G protein started to aggregate and many HSQC peaks became too broad to be detected. Further addition of ATPP immediately triggered visible precipitation and no NMR signal could be detected.

Furthermore, upon replacing the oxygen atom linking the beta and gamma phosphates with a carbon atom, the capacity of AMP-PCP (Fig. 1) to induce folding reduced to a level very similar to that of ADP: only at a ratio of 1:8 (C71G:AMP-PCP) the unfolded state was completely converted into the folded state (Supplementary Fig. 14b). On the other hand, unlike ADP which triggered no precipitation even at 20 mM, upon adding AMP-PCP to 1 mM, the C71G-hPFN1 protein started to aggregate and precipitate after one hour. Intriguingly, AMP-PNP with the oxygen atom replaced by a nitrogen atom also has the capacity to induce folding very similar to that of ADP and AMP-PNP (Supplementary Fig. 14c). However, its ability to trigger aggregation is even higher than that of AMP-PCP: at 1:10 (C71G:AMP-PNP) with the AMP-PNP concentration of only 0.5 mM, the C71G-hPFN1 protein started to precipitate after one hour.

### NMR characterization of the Zn^2+^-induced conformational equilibrium of hSOD1

Nascent hSOD1 is initiated to fold upon induction by Zn, which is coordinated by His63, His71, His80, and Asp83 (Supplementary Fig. 15a and S16a). Nascent hSOD1 is completely unfolded, characteristic of a narrowly-dispersed HSQC spectrum (Supplementary Fig. 15b). However, upon stepwise adding Zn2+, a folded population was formed as unambiguously indicated by the manifestation of a new set of well-dispersed HSQC peaks, which is largely saturated at 1:20 (hSOD1:Zn) where both folded and unfolded states still co-exist (Supplementary Fig. 15c).

For the folded state, many residues have large and negative (ΔCα–ΔCβ) chemical shifts (Supplementary Fig. 16b), indicating that they adopt well-formed β-strands. Most convincingly, except for several residues close to the mutation site C6 and C111, (ΔCα–ΔCβ) of the Zn^2+^-induced state of nascent WT-hSOD1 are highly similar to those of the Zn2+ -induced state of the pseudo-WT-hSOD1 C6A/C111S (Supplementary Figs. 16c and 16d). The results together suggest that in the presence of Zn^2+^, both WT and pseudo-WT hSOD1 adopt the highly similar β-barrel structures observed on the mature hSOD1 (Supplementary Fig. 16a). Nevertheless, upon being induced by Zn2+, the pseudo-WT-hSOD1 became completely folded, while the WT-hSOD1 still has a coexistence of the unfolded and folded states.

Moreover, most residues of the folded state have positive hNOE values with an average of 0.61, while some are even higher than 0.8, implying that the folded state has highly restricted backbone motions. Here we also collected HSQC-NOESY in the presence of Zn at 1:20. A large number of long-range NOEs has been observed, indicating that the folded state has well-packed tertiary structure. On the other hand, we found no NOE cross-peaks resulting from the exchange between two states, indicating that the time scale for the conformational exchange of two states of WT-hSOD1 in the presence of Zn at 1:20 is slower than that of C71G-hPFN1. In other words, the energy barrier separating two states of WT-hSOD1 is larger than that separating two states of C71G-hPFN1^43^. As such, we were unable to characterize its exchange dynamics as conducted for C71G-hPFN1.

### ATP and triphosphate induce the folding of nascent hSOD1

**Fig. 4:**
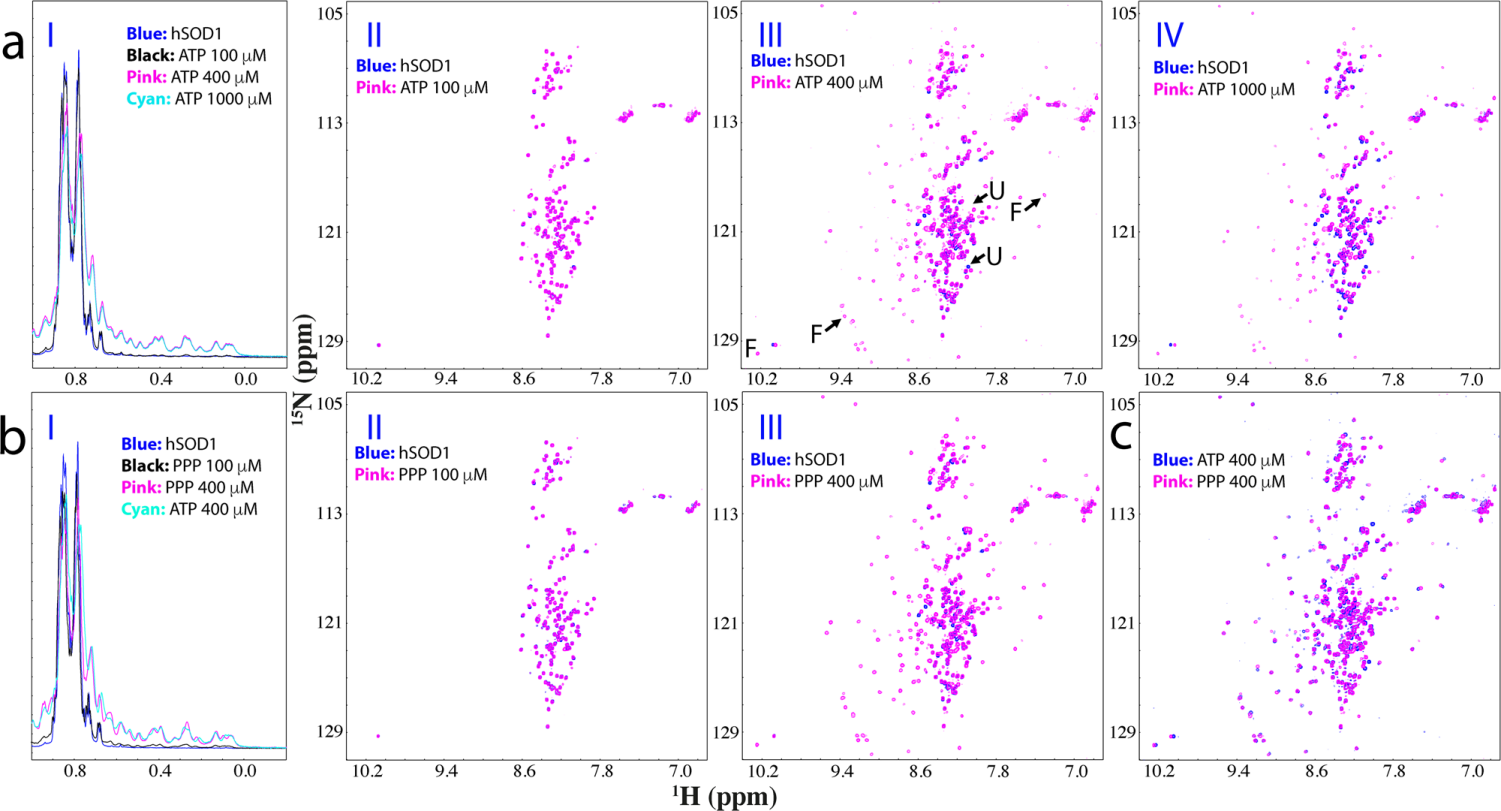
ATP or triphosphate induces folding of the unfolded nascent hSOD1. **a** Up-field 1D NMR spectra and superimposition of HSQC spectra of ^15^N-labeled nascent hSOD1 at a concentration of 50 μM in the absence (blue) or in the presence of ATP (pink) at different molar ratios. Some characteristic NMR signals of the folded (F) and unfolded (U) states were indicated by arrows. **b** Up-field 1D NMR spectra and superimposition of HSQC spectra of nascent hSOD1 at a concentration of 50 μM in the absence (blue) or in the presence of Triphosphate (pink) at different molar ratios. **c** Superimposition of HSQC spectra of nascent hSOD1in the presence of ATP at 1:2 (blue) and PPP (pink) at 1:2.

Here, nascent hSOD1 adopting the distinct Greek-key β-barrel fold provides an excellent model for addressing whether ATP can induce folding from the completely unfolded state. Therefore, we conducted titrations of ATP into the nascent hSOD1 sample as monitored by NMR 1D and HSQC spectroscopy (Fig. 4a). Upon adding ATP to 1:2, no significant change was observed on 1D (I of Fig. 4a) and HSQC spectra (II of Fig. 4a). Unexpectedly, when the ratio was increased to 1:8, well-dispersed HSQC peaks and very up-field 1D signals manifested (III of Fig. 4a), as well as further evidenced by the intensity changes at different ratios (Supplementary Fig. 17). Further addition of ATP even up to 1:20 only led to some slight shifts of HSQC peaks of the unfolded state (IV of Fig. 4a) but up-field 1D peaks from the folded state at 1:20 is highly similar to that at 1:8 only with the intensity slightly reduced (I of Fig. 4a). However, the addition of ATP beyond 1:20 triggered visible precipitation and resulted in disappearance of NMR signals. The results unambiguously indicate that ATP could also induce folding of the completely unfolded nascent hSOD1, and the induction was largely saturated at 1:8. On the other hand, like Zn^2+^ ATP could not completely convert the unfolded state into the folded states.

To assess which group of ATP has the inducing capacity, we first titrated nascent hSOD1 with adenosine but even with the highest concentrations of 5 mM, no large shift of HSQC peaks and no manifestation of up-field 1D signals were detected (Supplementary Fig. 18), indicating no ability to induce folding. We further titrated sodium triphosphate (PPP) into nascent hSOD1 (Fig. 4b). Indeed, PPP could also induce the folding of the unfolded nascent hSOD1. Briefly, very similar to what was observed on ATP, at 1:2, no significant change was observed on 1D (I of Fig. 4b) and HSQC (II of Fig. 4b) spectra. However, from 1:4 the folded population was formed with the manifestation of the well-dispersed HSQC and up-field 1D peaks. From 1:6 to 1:8, only a slight increase in the peak intensity of the folded state and a reduction in the peak intensity of the unfolded state (Supplementary Fig. 19). Evidently, at 1:8 1D spectra of ATP and PPP were very similar (I of Fig. 4b), while most HSQC peaks were also superimposable (Fig. 4c), thus indicating that the capacity of ATP to induce folding of nascent hSOD1 also come from its triphosphate group. However, further addition of PPP at 1:10 triggered visible precipitation and resulted in the disappearance of NMR signals.

We further conducted the titrations with ADP but no folded state was induced even with a ratio up to 1:40 (Supplementary Fig. 20) above which the sample started to precipitate. Subsequently, we also titrated with sodium pyrophosphate and no induction of folding was detected with the ratio of 1:10 above which the NMR sample started to precipitate. Moreover, in our previous studies on hSOD1^31,32,53^, we have systematically assessed the effects of various salts with different cations and anions. Except for Fe^2+^, none of them could induce folding of nascent hSOD1 but all induced aggregation at high concentrations.

So why do ATP and triphosphate have the very similar capacity in inducing folding, but different abilities in triggering aggregation? Structurally, ATP is composed of adenosine and anionic triphosphate group, which is highly negatively charged and thus can exert a strong electrostatic screening effect. In this context, for proteins such as C71G-hPFN1 and nascent hSOD1 which are partially- or highly-disordered with considerable hydrophobic patches exposed, triphosphate not only induces folding but simultaneously imposes a screening effect, which leads to aggregation mainly driven by hydrophobic interaction^11^.

Interestingly, once triphosphate is covalently linked to adenosine to form ATP, on the one hand, ATP still owns the capacity of triphosphate to induce folding but on the other hand its screening effect to trigger the aggregation appears to be reduced to some degree. This might result from at least two possible mechanisms: (1) the screening effect of triphosphate in ATP is attenuated to some degree by interacting with adenosine; or/and (2) the aromatic base ring of adenine in ATP may dynamically interact with the exposed hydrophobic patches of proteins, thus serving to shield the hydrophobic patches from the screening effect of triphosphate.

### Comparison of conformations and dynamics of ATP− and Zn^2+^-induced folded states

**Fig. 5:**
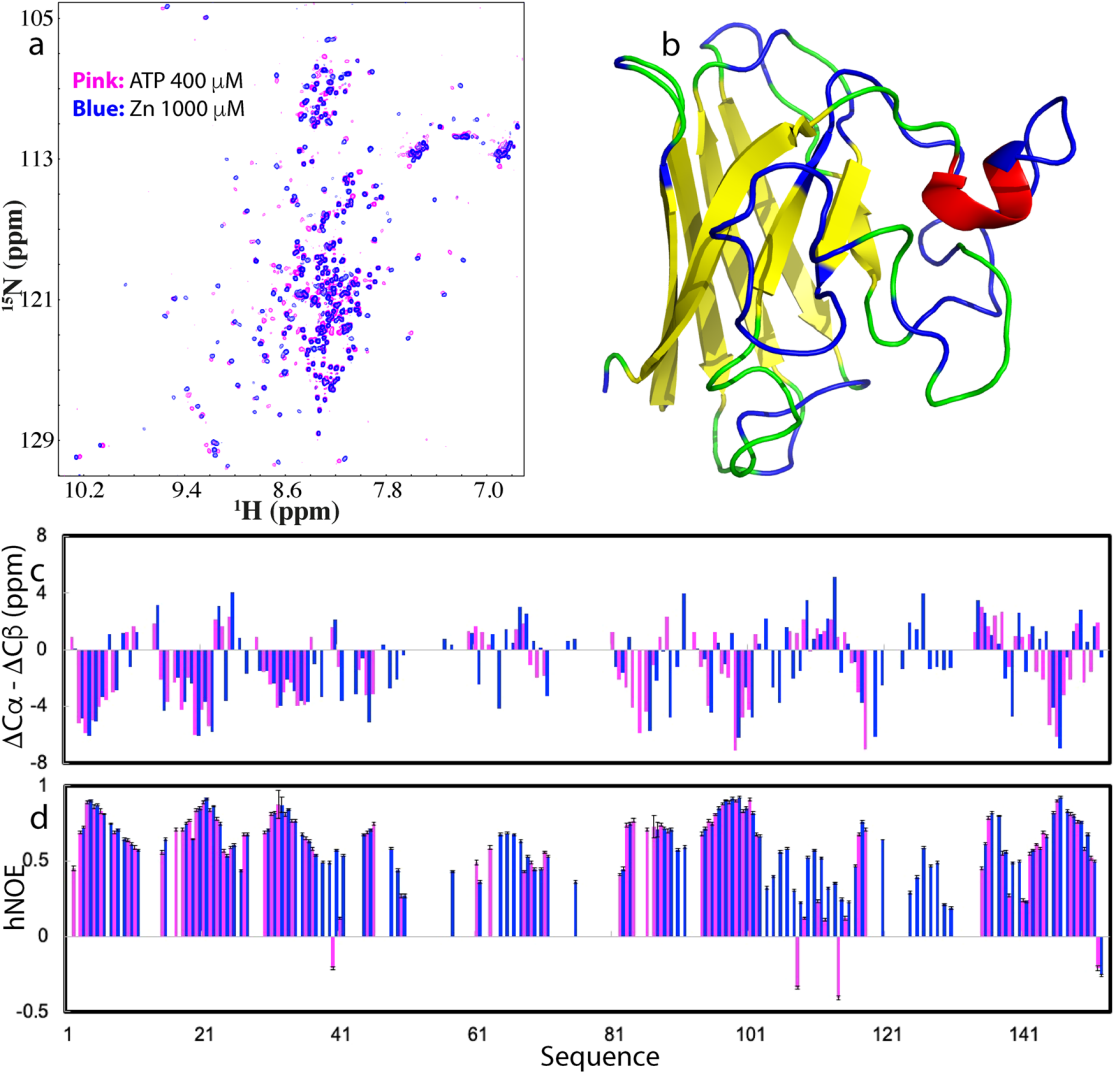
Comparison of conformations and dynamics of the ATP- and Zn-induced folded state. **a** Superimposition of HSQC spectra of ^15^N-labeled hSOD1 in the presence of ATP at 1:8 (blue) and in the presence of zinc cation (pink) at 1:20. **b** NMR structure of C6A/C111S with the unassigned residues of the ATP-induced folded state colored in blue due to missing or overlapping resonances. **c** Residue specific (ΔCα–ΔCβ) chemical shifts of the ATP-induced (purple) and Zn^2+^-induced (blue) folded states of hSOD1. **d** {^1^H}–^15^N heteronuclear steady-state NOE (hNOE) of the ATP-induced (purple) and Zn^2+^-induced (blue) folded states of hSOD1.

To compare the solution conformations and dynamics of ATP− and Zn^2+^-induced folded states, we superimposed their HSQC spectra acquired at the same protein concentrations and buffer conditions (Fig. 5a). While many HSQC peaks of two states were highly superimposable, some have relatively large differences. Thus, we further collected and analyzed a set of triple-resonance NMR spectra of the ATP-induced state for sequential assignment. Intriguingly, for some residues, although they had detectable HSQC peaks, their Cα and Cβ resonance peaks were undetectable in the triple-resonance experiments. This is reminiscent of what we previously observed on a 38-residue WW1 domain whose HSQC peaks were detectable but no Cα and Cβ peaks were observed for the majority of WW1 residues in HNCACB and CBCANH spectra, implying that these residues might undergo conformational exchanges on μs-ms time scale^54^.

The analysis of triple-resonance spectra led to the assignments of the residues of the ATP-induced folded state of hSOD1 except for 57 residues, which include Ala1, Lys9, Gly12, Asn26-Gly27, Leu38-Thr39, Leu42-His43, Val47-Ala60, Phe64-Asn65. Gly72-His80, Asp90-Asp92, Val103-Leu106, Val119-Glu133, Gln153 (Fig. 5b). Most unassigned residues are located over the loop regions, particularly for those involved in forming the Zn-binding pocket. For the assigned residues, most of them have (ΔCα–ΔCβ) chemical shifts very similar to those corresponding residues of the Zn-induced state (Fig. 5c). The results suggest that the β-strands of the characteristic β-barrel structure of the mature hSOD1 have been already well-formed in the ATP-induced state. However, due to the lack of Zn, the loop regions constituting the binding pocket of Zn appeared to still undergo intermediate conformational exchanges and thus became too broad to be undetectable.

We also acquired {^1^H}–^15^N hNOE data of the ATP-induced folded state with an average of 0.59, only slightly smaller than that of the Zn-induced state (0.61) (Fig. 5d). Briefly, the residues of the β-strands have hNOE values very similar to those corresponding residues of the Zn-induced state. However, Glu40, Gly108, and Gly114 have negative hNOE of −0.21, −0.34 and −0.41 respectively. This result indicates that without the Zn-coordination, the residues around the Zn-binding pocket have conformational dynamics on both ps-ns and μs-ms time scales. Furthermore, we have collected HSQC-NOESY spectrum on the sample of the ATP-induced state and like the Zn-induced state, no NOE cross-peak was identified to result from the exchange between the unfolded and folded states, indicating that like the Zn-induced state, the energy barrier separating two states of hSOD1 induced by ATP is also larger than that of C71G-hPFN1.

**Fig. 6:**
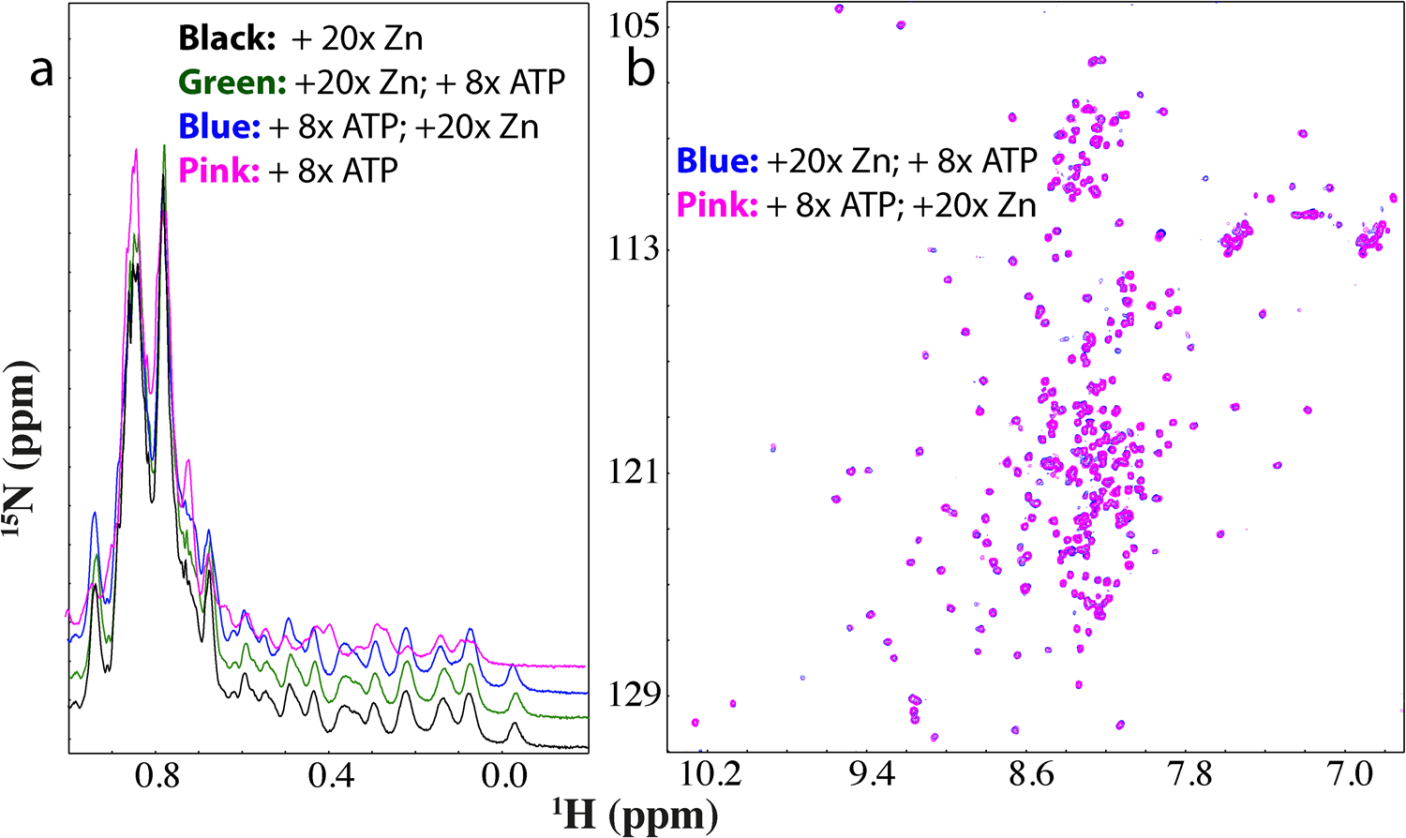
ATP and Zn interplay in inducing folding of nascent hSOD1. **a** Up-field 1D NMR spectra of hSOD1 in the presence of zinc at 1:20 (black) and with further addition of ATP at 1:8 (green) as well as in the presence of ATP at 1:8 (purple) and with further addition of zinc at 1:20 (blue). **b** Superimposition of HSQC spectra of ^15^N-labeled hSOD1 in the presence of zinc at 1:20 with further addition of ATP at 1:8 (blue) and in the presence of ATP at 1:8 with further addition of zinc at 1:20 (purple).

We also attempted to assess whether ATP and zinc together can completely convert the unfolded population into the folded state either by adding ATP into the hSOD1 sample with the pre-existence of Zn at 1:20, or adding Zn into the hSOD1 sample with the pre-existence of ATP at 1:8. Interestingly, both up-field 1D (Fig. 6a) and HSQC (Fig. 6b) peaks of two resulting hSOD1 samples became very similar, as well as similar to those of hSOD1 sample only in the presence of zinc at 1:20. The results further confirm that the major difference between ATP- and Zn-induced folded states are over the loop regions as reflected by (ΔCα–ΔCβ) chemical shifts (Fig. 5c). Furthermore, in 3D HSQC-NOESY spectrum of the hSOD1 sample in the presence of ATP at 1:8 and Zn at 1:20, again no NOE cross-peak was identified to result from the exchange between the unfolded and folded states, implying that even in the presence of both ATP and Zn, the two states are still separated by the energy barrier larger than that of C71G-hPFN1.

### TMAO showed no detectable induction of folding of C71G-hPFN1 and nascent hSOD1

**Fig. 7:**
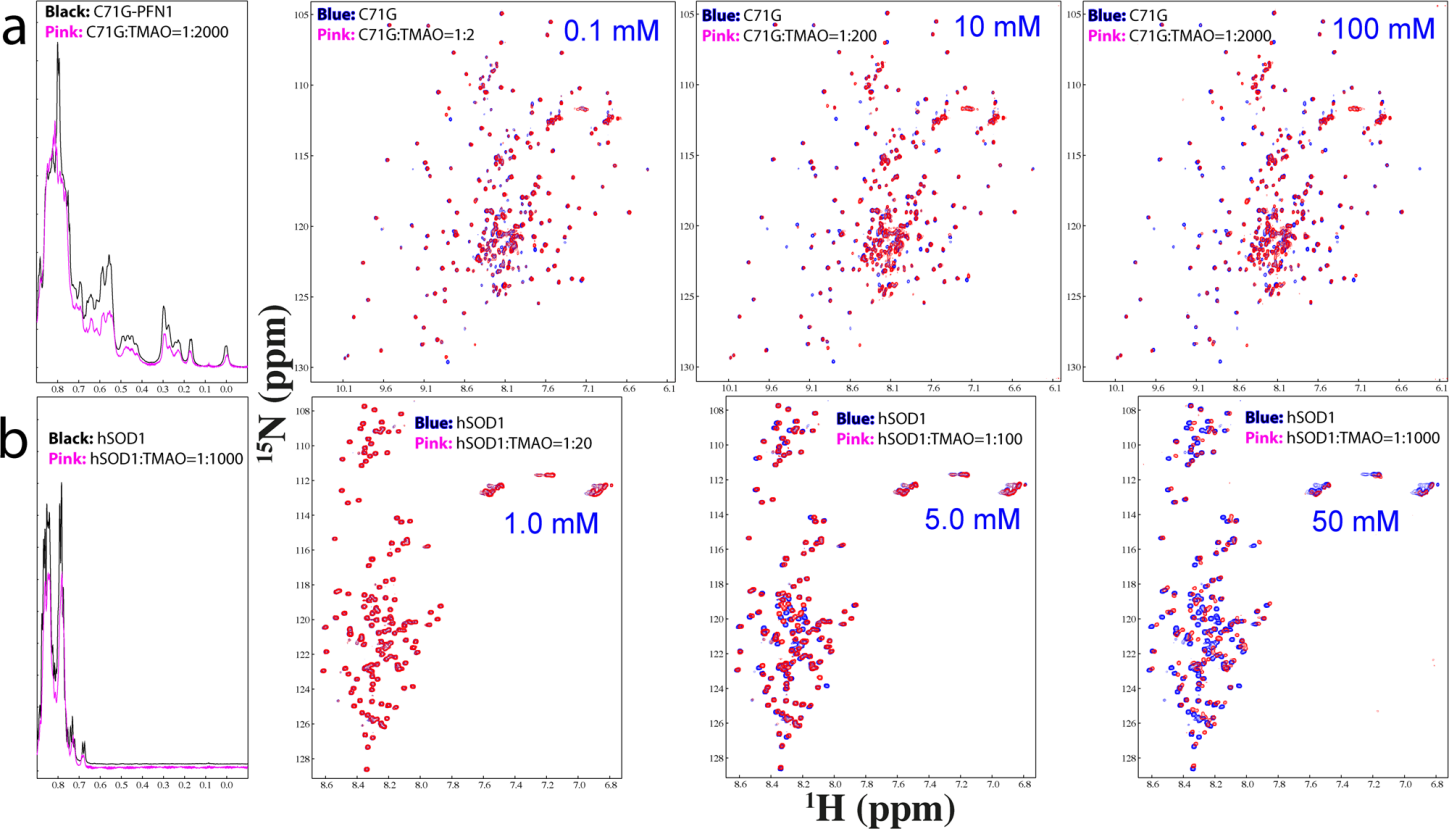
TMAO showed no capacity in inducing folding of C71G-hPFN1 and hSOD1. **a** Up-field 1D and HSQC spectra of C71G-hPFN1 at a concentration of 50 μM in the presence of TMAO at molar ratios up to 1:2000 (C71G:TMAO) at which TMAO triggered a complete precipitation. For clarity, only 1D spectra of C71G-hPFN1 in the free state (black) and in the presence of TMAO at 1:2000 were presented. **b** Up-field 1D and HSQC spectra of hSOD1 at a concentration of 50 μM in the presence of TMAO at molar ratios up to 1:1000 (hSOD1:TMAO) at which TMAO triggered a complete precipitation.

Currently, the best-known molecule with the general capacity to induce protein folding is the natural osmolyte, trimethylamine N-oxide (TMAO)^3,55–57^. Here, we titrated TMAO into the C71G-hPFN1 sample (Fig. 7a). At 1:2 (C61G:TMAO), except for the shift of several peaks, no large changes were observed for HSQC peaks of both folded and unfolded states. At 1:200, shifts were observed for HSQC peaks of both folded and unfolded states. However, even at 1:2000, HSQC peaks of both folded and unfolded states still coexisted but the intensity of 1D peaks for both states reduced considerably. Moreover, at 1:2000, the C71G-hPFN1 sample becomes completely precipitated after one hour. The results clearly revealed that TMAO was unable to convert the unfolded state of C71G-hPFN1 into the folded state even with a ratio up to 1:2000.

Subsequently, we also titrated TMAO into a nascent hSOD1 sample. As shown in Fig. 7b, at 1:20, no large shift was observed for HSQC peaks of nascent hSOD1. At 1:100, many peaks showed large shifts but no well-dispersed peaks manifested. Even at 1:1000, the majority of HSQC peaks underwent large shifts but no up-field 1D and well-dispersed HSQC peaks manifested. However, at 1:1000, NMR peaks became broad and the sample became completely precipitated after one hour.

The results unambiguously revealed that, unlike ATP and ADP, TMAO failed to convert C71G-hPFN1 into the folded state, while, unlike ATP and Zn^2+^, TMAO was unable to induce the formation of any folded population. This is in general consistent with previous reports that TMAO concentrations needed to reach >M to induce the folding of proteins (3,556,56). However, for diseases-causing proteins highly-prone to aggregation, at such high concentrations, the polar TMAO molecules could also induce aggregation.

### ATP enhances thermodynamic stability of C71G- but not WT-hPFN1

**Fig. 8:**
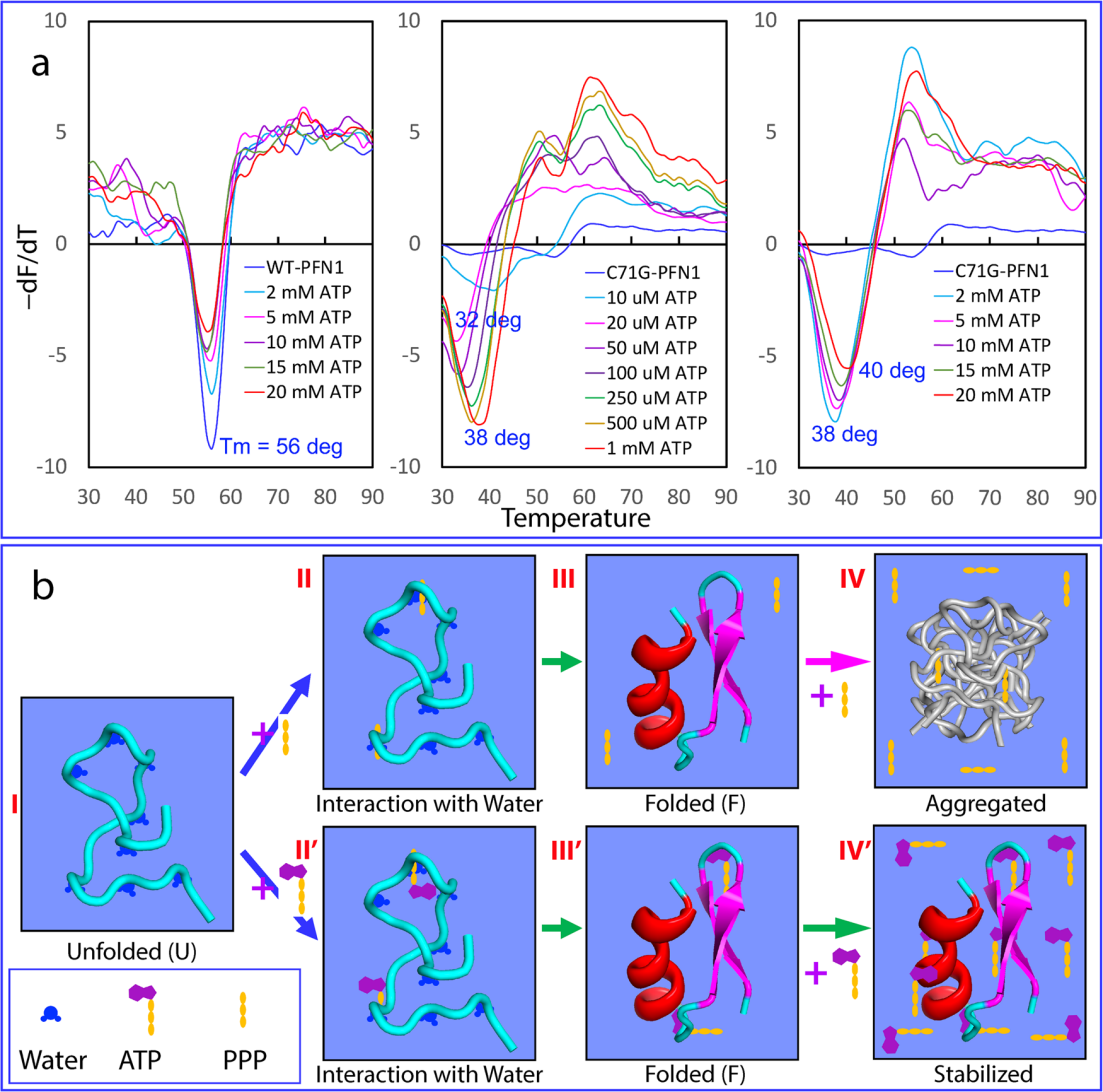
The speculative mechanism for ATP and PPP to induce protein folding. **a** ATP enhances thermodynamic stability of C71G-hPFN1 but not WT-hPFN1 as measured by differential scanning fluorimetric (DSF). Melting curves of thermal unfolding of WT-hPFN1 and C71G-hPFN1 in the presence of ATP at different concentrations by plotting the first derivative of the fluorescence emission as a function of temperature (−dF/dT). Here Tm is represented as the lowest point of the curve. **b** A speculative model for protein folding induced by triphosphate (PPP) and ATP. For the unfolded state (U) of a protein, many backbone atoms are hydrogen-bonded with water molecules. Both triphosphate (PPP) and ATP can use their triphosphate chain to effectively attract water molecules out from hydrogen-bonding with the backbone atoms, thus favoring the formation of the folded state (F). However, due to the electrostatic screening effects produced by highly charged triphosphate, a protein exemplified by C71G-hPFN1 and nascent hSOD1 with significant exposure of hydrophobic patches due to the defects in tertiary packing will become severely aggregated. By contrast, ATP can use its aromatic base ring to dynamically interact with the exposed hydrophobic patches, which consequently not only functions to prevent aggregation but also to increase the thermodynamic stability.

We also measured the thermodynamic stability by differential scanning fluorimetric (DSF) method^21–23,58^. Interestingly, WT-hPFN1 has a melting temperature (Tm) of 56 °C while the addition of ATP even up to 20 mM triggered no significant change (Fig. 8a). By contrast, C71G-hPFN1 without ATP has no cooperative unfolding signal (Fig. 8a), most likely due to the absence of tight tertiary packing or/and coexistence of two states^59,60^. However, in the presence of ATP of 20 μM (a molar ratio of 1:2), a cooperative unfolding signal was observed with Tm of 32 °C. The addition of ATP to 1 mM (1:100) led to the increase of Tm to 38 °C, and further increase to 40 °C at 20 mM (Fig. 8a).

We also performed DSF measurements of C71G-hPFN1 with the addition of PPP at various ratios but only obtained curves with high noises and no cooperative unfolding signals, implying that although ATP and PPP both have similar capacity in inducing folding, PPP failed to enhance tight tertiary packing of the folded state or/and triggered dynamic aggregation even before the visible precipitation. Indeed, previously we found that a small protein could still have native-like NMR spectra but its tight packing was disrupted to different degrees^59,60^. We also performed DSF measurements of hSOD1 with Zn^2+^ or/and ATP at various ratios but obtained curves with high noises and no cooperative unfolding signals, most likely due to the absence of tight tertiary packing or/and coexistence of two states.

## Discussion

Protein misfolding/aggregation is associated with an increasing spectrum of human diseases, including neurodegenerative diseases^9,11^, and aging down to *E. coli* cells, yet the underlying mechanism remains a great mystery^9–11,61–63^. While hPFN1 and hSOD1 have distinct functions and structures (Fig. 1b), by a gain of toxicity through misfolding/aggregation, C71G-hPFN1 causes FALS and nascent hSOD1 is associated with SALS. Therefore, defining dynamic/thermodynamic properties and mechanisms, as well as identifying small molecules that modulate their folding/misfolding/aggregation, not only offers fundamental insights into ALS pathogenesis, but may pave the way for developing therapeutic agents to treat ALS and other aggregation-related diseases and aging.

In this study, we successfully quantified the populations of folded and unfolded states of C71G-hPFN1 respectively as 55.2% and 44.8%, exchanging at 11.7 Hz. Although the folded state of C71G-hPFN1 has a conformation similar to WT-hPFN1, it exhibits increased backbone flexibility on the ps-ns time scale, and its thermodynamic stability is significantly lower than that of WT-hPFN1. These findings elucidate the biophysical mechanism for the high propensity of C71G-hPFN1 to misfold and aggregate both in vitro and in vivo. The relatively small energy barrier between its folded and unfolded states causes rapid conversion of the folded state into the unfolded state upon misfolding/aggregation of the latter, resulting in eventual misfolding/aggregation of all C71G-hPFN1 proteins. Additionally, even the folded state may directly undergo misfolding/aggregation due to its reduced dynamic and thermodynamic stability.

For hSOD1, recently through a systematic assessment, we made a surprising discovery. Except for Zn^2+^, Cu^2+^ and Fe^2+^, 12 cations (Na^+^, K^+^, Ca^2+^, Zn^2+^, Mg^2+^, Mn^2+^, Cu^2+^, Fe^2+^, Ni^2+^, Cd^2+^, Co^2+^ and Al^3+^) lack the Zn^2+^-like ability to bind and induce the initial folding of nascent hSOD1^53^. This finding suggests that nature specifically selects Zn^2+^ to serve as the cofactor for hSOD1, not just because it is a positively charged cation, but due to its unique integration of at least three abilities: (1) to specifically coordinate the formation of the Zn^2+^-binding pocket; (2) to induce the initial folding of nascent hSOD1 into a coexistence of two states; and (3) to occupy the pocket of mature hSOD1, serving as a cofactor for enzymatic functions. This discovery emphasizes the critical role of Zn^2+^ in switching toxic nascent hSOD1 into the non-toxic folded state, providing an explanation for in-cell results^64^ and suggesting that increasing effective cellular concentrations of zinc, for example, through dietary intake, could be an important therapeutic approach for treating SALS patients caused by WT hSOD1^65^.

Most unexpectedly, our present study revealed that ATP can convert C71G-hPFN1 into the folded population and induce the co-existence of the folded and unfolded states of hSOD1, at respective ratios of 1:2 and 1:8. In contrast, TMAO showed no detectable induction for C71G-hPFN1 and nascent hSOD1, even at much higher ratios. Remarkably, we found that the inducing capacity of ATP comes from its triphosphate moiety, which was previously proposed to act as a crucial intermediate in prebiotic chemical evolutions, generating building units for constructing primitive cells and potentially contributing to the Origin of Life^66,67^. Furthermore, the inducing capacity of 10 ATP-related molecules was ranked as: ATP = ATPP = PPP > ADP = AMP-PNP = AMP-PCP = PP, while AMP, adenosine, P, and NaCl showed no conversion before aggregation. The results also indicate that the inducing capacity depends not only on the number of phosphate groups but also on the atoms linking the phosphate groups. The linkage of adenosine and triphosphate represents the best combination, retaining the capacity of triphosphate to induce folding but minimizing its potential to trigger aggregation.

The comparison of NMR conformations and dynamics of the ATP− and Zn^2+^-induced folded states indicates that ATP/triphosphate and Zn^2+^ induce folding of nascent hSOD1 by fundamentally-different mechanisms: Zn^2+^ induces folding by providing the specific information additional to the amino acid sequence, while ATP and triphosphate act to induce folding by generally enhancing the intrinsic folding capacity encoded by the sequence. For hSOD1, its Cys residues appear to carry crucial information that determines the energy landscape for its maturation. In the absence of the disulfide bridge, ATP or Zn^2+^ is unable to fully convert the unfolded state into the folded state, likely due to a larger energy barrier separating the two states compared to C71G-hPFN1. Notably, in the super-stable C6A/C111S mutant, where Cys is mutated to Ser and Ala, the barrier is reduced or eliminated, resulting in complete folding upon induction by Zn^2+^ without any unfolded population^34^. This observation is of great significance in understanding protein folding, suggesting that kinetic barriers associated with Cys residues might play a key role in the maturation process of hSOD1, similar to other proteins like Cathepsin B^68,69^. Interestingly, mature Cathepsin B, which lacks its propeptide essential for initial folding, loses the ability to spontaneously refold back to its native state and becomes precipitated when unfolded. However, when the active-site Cys29 residue is mutated to Ser, the mature Cathepsin B mutant can suddenly refold back to its native structure after unfolding, indicating the critical role of Cys29 in constituting the kinetic barrier during its folding process^69^.

So, what could be the mechanism for ATP and triphosphate to generally induce folding respectively at 1:2 and 1:8 for C71G-hPFN1 and hSOD1, which have unrelated functions and structures? At such low ratios, unlike what was proposed for TMAO with concentrations >M^3,55–57^, ATP is not anticipated to induce folding by the volume-excluding effect. After exhaustively reviewing the literature on the mechanisms of protein folding, we believe that the most relevant explanation is that ATP and triphosphate act to induce folding by interacting with protein hydration. Previously, it was proposed that hydrogen bonding with water molecules or/and solvation, particularly for the protein backbone atoms play a key role in protein folding^2–4^. The unfolded state will be favored if the backbone atoms are highly hydrogen-bonded with water molecules, while the folded state will be favored if the backbone atoms are involved in forming intramolecular hydrogen-bonds. Indeed, our previous NMR studies suggest that ATP and triphosphate have a high capacity in mediating the hydration of proteins even without needing strong and specific binding^17,19,20,23^. Recent NMR and MD simulation results also indicate that in general for proteins without the ability to bind nucleic acids, ATP only has a very weak and non-specific effect on folded proteins even at very high ATP/protein ratios, which, however, is sufficient to alter protein hydration^70^.

In this context, we propose that ATP and triphosphate induce folding mainly by interacting with and displacing water molecules that hydrogen-bond with the unfolded protein (Fig. 8b). Briefly, the unfolded protein (I of Fig. 8b) is hydrogen-bonded with multiple water molecules. However, in the presence of triphosphate or ATP, some of these water molecules switch their interactions to triphosphate (II of Fig. 8b) or ATP (II’ of Fig. 8b), leading to the folding of the protein (III and III’ of Fig. 8b). Nevertheless, further addition of triphosphate induces protein aggregation due to the strong screening effect of triphosphate (IV of Fig. 8b). In contrast, further addition of ATP enhances the thermodynamic stability of the protein by dynamically interacting with and shielding hydrophobic patches from exposure to the bulk solvent (IV’ of Fig. 8b). In this context, ATP and triphosphate induce folding by enhancing the intrinsic folding capacity already encoded in their sequence. Indeed, even at 1:400, ATP showed no induction of folding of intrinsically-disordered prion-like and RGG-rich domains of FUS^19^ and TDP-43^20^ as these sequences intrinsically encode no folded structures. On the other hand, the results with ATP and its analogs decode that extremely specific structural features are required to possess the capacity to induce protein folding, which is not only determined by the number of phosphates but even by the atoms linking the phosphates.

In summary, nature appears to select triphosphate as the central intermediate for prebiotic evolution also because of its remarkably high efficiency in inducing protein folding. However, likely because the high concentrations of triphosphate inevitably trigger aggregation of unfolded or partially-unfolded protein states, triphosphate concentrations are extremely low in most modern cells. As ATP induces folding through interactions other than the screening effect, its inducing capacity should remain unaffected in cellular environments, even at salt concentrations equivalent to ~150 mM NaCl. Evidently, as shown in Fig. 8a, the increase of ATP concentrations not only failed to screen out its inducing effect but instead constantly enhanced the thermodynamic stability of C71G-hPFN1, although ATP is also a highly-charged salt itself. In particular, the ionic strength of ATP at 20 mM is higher than that of 150 mM NaCl. Indeed, in some single-cell organisms, inorganic polyphosphates function as primordial chaperones^45,46^. Here we propose that polyphosphates might share the same mechanism of triphosphate to generally facilitate protein folding. In this context, ATP may have played a central role in solving protein folding and aggregation challenges in primitive cells lacking modern ATP-energy-dependent machinery, thus being irreplaceable for the Origin of Life. Even in modern cells, ATP appears to still operate at fundamental levels to energy-independently prevent misfolding/aggregation by enhancing the intrinsic folding capacity of proteins. This may shed light on the long-standing enigma of age-related onset of FALS and increased risk of other neurodegenerative diseases, as cellular ATP concentrations decrease with aging^71^. In the future, it is of significant interest to devise experimental and computational strategies to explore the energy-independent functions of ATP in living cells with elevated salt concentrations and macromolecular crowding. These efforts may pave the way for engineering molecules with ATP-like properties that efficiently induce folding, inhibit aggregation, and increase the stability of proteins for treating aggregation-associated diseases and other applications.

## Methods

### Chemicals and optimization of buffer conditions

ATP, ADP, AMP, sodium tripolyphosphate (PPP), sodium pyrophosphate (PP), ATPP, AMP-PCP, AMP-PNP, Adenosine, sodium phosphate, sodium chloride, zinc acetate and trimethylamine N-oxide (TMAO) were all purchased from Sigma-Aldrich. The fluorescent dye SYPRO Orange (S5692–50UL) was purchased from Sigma-Aldrich^22,23^.

Like all proteins associated with neurodegenerative diseases, C71G-hPFN1, and nascent hSOD1 are highly aggregation-prone both in cells and in buffers with high salt concentrations^11^. Here, based on our previous results from exhaustive screenings of buffer conditions including pH, types, and concentrations of salts^31,32,41^, we further optimized the buffers for the current study on C71G-hPFN1 and hSOD1, in order to minimize their aggregation to allow time-consuming high-resolution NMR characterization.

In our previous study^41^, we systematically evaluated the effects of pH and salts including NH_4_Cl, NaCl, KCl, Zn Cl_2_, CaCl_2_, and MgCl_2_ on the conformations and aggregation of C71G-hPFN1. The results indicated that: (1) at pH above 6.5, considerable aggregation occurred; (2) no such cation or anion had any detectable ability to induce folding but all triggered precipitation at high concentrations. Therefore, here C71G-hPFN1 as well as ATP related compounds (Fig. 1a) were all prepared in 1 mM phosphate buffer containing 2 mM DTT with their final pH adjusted to pH 6.0 by diluted NaOH or H_3_PO_4_. On the other hand, hSOD1 of the native sequence contains four cysteine residues and started to form intermolecular disulfide bridges to lead to rapid precipitation even in the presence of 10 mM DTT once the solution pH was above 5.0^31,32^. As such, nascent hSOD1 as well as zinc acetate and ATP related compounds were all prepared in 1 mM acetate buffer with their final pH adjusted to pH 4.5 by diluted NaOH or HAc.

### Preparation of WT-/C71G-hPFN1 and hSOD1 proteins

The expression and purification of WT-hPFN1 and C71G-hPFN1, as well as the removal of His-tag followed the previous protocol^41^. On the other hand, The gene encoding the wild-type hSOD1 of the native sequence was purchased from Genscript with *E. coli* preferred codons. To remove the inference of extra residues, the gene was subsequently cloned into a modified vector pET28a without any tag^31,32^. Then the expression vector was transformed into and overexpressed in Escherichia coli BL21 (DE3) cells (Novagen). The recombinant hSOD1 protein was found in the inclusion body. As a result, the pellets were first washed with buffers several times and then dissolved in a phosphate buffer (pH 8.5) containing 8 M urea and 100 mM dithiothreitol (DTT) to ensure complete conversion to Cys-SH. After 1 h, the solution was acidified by adding 10% acetic acid and subsequently purified by reverse-phase (RP) HPLC on a C4 column eluted by water-acetonitrile solvent system^15,16^. The HPLC elution containing pure recombinant hSOD1 was lyophilized and stored at −80 degrees. When the protein powders were dissolved in the buffers, C71G-hPFN1 became co-existing with the two states, while hSOD1 remained unfolded.

To generate isotope-labeled WT-/C71G-hPFN1 and hSOD1 proteins for NMR studies, the bacteria were grown in M9 medium with the addition of (^15^NH_4_)_2_SO_4_ and ^13^-Glucose for ^15^N-/^13^C-labeling^27^. The protein concentrations were determined by the UV spectroscopic method in the presence of 8 M urea, under which the extinct coefficient at 280 nm of a protein can be calculated by adding up the contribution of Trp, Tyr, and Cys residues^31,32,41,72^.

### NMR titrations

All NMR experiments were acquired at 25 °C on an 800 MHz Bruker Avance spectrometer equipped with pulse field gradient units and a shielded cryoprobe^31,32,41,44,49^. To conduct NMR titrations of ATP, ADP, AMP, ATPP, AMP-PCP, AMP-PNP, Adenosine, PPP, PP, sodium phosphate, sodium chloride, and TMAO, two-dimensional ^1^H-^15^N NMR HSQC spectra were collected on the ^15^N-labeled C71G-hPFN1 at a protein concentration of 50 μM in 1 mM sodium phosphate buffer containing 2 mM DTT (with the final pH adjusted to 6.0) at 25 °C in the presence of ATP, ADP and AMP at molar ratios of 1:0.25, 1:0.5, 1:1, 1:2, 1:4, 1:6, 1:8, 1:10, 1:15, 1:20, 1:40, 1:100, 1:200, 1:300 and 1:400. For Adenosine, the ratios are 1:0.25, 1:0.5, 1:1, 1:2, 1:4, 1:6, 1:8, 1:10, 1:15, 1:20, 1:40, 1:100. For PPP and ATPP, the ratios are 1:0.25, 1:0.5, 1:1, 1:2, 1:4 where the sample started to precipitate. For PP, AMP-PCP, and AMP-PNP, the ratios are 1:0.25, 1:0.5, 1:1, 1:2, 1:4, 1:6, 1:8, 1:10 where the sample started to precipitate. For sodium phosphate, the ratios are 1:1, 1:2, 1:10, 1:20, 1:40, 1:100 where the sample started to precipitate. For sodium chloride, the ratios are 1:1, 1:10, 1:20, 1:40, 1:100, and 1:200 where the sample started to precipitate.

For NMR titration of zinc acetate, ^15^N-labelled hSOD1 samples at a protein concentration of 50 μM in 1 mM sodium acetate-d3 buffer at pH 4.5 were used with stepwise additions of zinc acetate at molar ratios of 1:0.5, 1:1, 1:2, 1:4, 1:6, 1:8, 1:10, 1:12, 1:16, 1:20, 1:24, 1:30 and 1:40. For NMR titrations of ATP and related molecules, ^15^N-labelled hSOD1 samples at a protein concentration of 50 μM were used with stepwise additions of ATP/PPP at molar ratios of 1:0.5, 1:1, 1:2, 1:4, 1:8, 1:12, 1:20, 1:24 and 1:30. For Adenosine, ^15^N-labelled hSOD1 samples at a protein concentration of 50 μM were used with stepwise additions of 1:0.5, 1:8, 1:20, 1:40, 1:60 and 1:100. For ADP/PP, 1:0.5, 1:4, 1:8, 1:12, 1:20, 1:30, 1:40 and 1:50.

NMR spectra were processed with NMR Pipe^73^ and analyzed with NMR View^74^. The molar ionic strength of the sodium salts was calculated^15,19,52^.

### NMR sequential assignments

To achieve sequential assignments of both WT-hPFN1 and C71G-hPFN1, triple resonance NMR spectra HNCACB and CBCANH were collected on the ^15^N-/^13^C- double labeled WT-/C71G-hPFN1 and hSOD1 (with zinc at 1:20 or ATP at 1:8) samples while HSQC-TOCSY and HSQC-NOESY were collected on the ^15^N-labeled samples. NMR ^1^H chemical shifts were referenced to external DSS at 0.0 ppm^31,32,41,44,49^.

### PGF diffusion measurement

PGF-NMR experiments were run on an 800 MHz Bruker Avance spectrometer at 25 °C. The different protein samples were prepared in D_2_O at a protein concentration of 50 µM in 1 mM sodium phosphate buffer (pD 6.0). The experiments were performed using the Bruker pulse sequence and the Bruker macro diffusion ordered spectroscopy (DOSY)^44,51^. Typically 16 values of gradient strength were used in the range 0 to 32 G/cm, with PFG duration of 2 ms, and diffusion time of 150 ms. The self-diffusion coefficients (*D*_*s*_) were calculated using the Bruker DOSY analysis program. Each sample was run in triplicate and *Ds* values were averaged over the three experiments. The resulting decay curves were fitted and *Ds* values were calculated with the equation below:

I = I(0)exp[-D(γgδ)^2^(Δ-(δ/3))]

Where I(0) is 1.002, γ is 4.258 × 10^3^ Hz/G, δ is 4.000 ms, and Δ is 150 ms.

### Quantification of exchange kinetics

Longitudinal magnetization transfer due to chemical exchange is the basis for the appearance of exchange cross peaks in nuclear Overhauser effect spectroscopy (NOESY) and for two-dimensional ZZ-exchange spectroscopy^43,75–79^. The evolution of longitudinal magnetization is described by:

$$\frac{d}{{dt}}\left[\begin{array}{c}\Delta {M}_{{zA}}(t)\\ \Delta {M}_{{zB}}(t)\end{array}\right]=\left[\begin{array}{cc}-{R}_{1A}^{0}-{p}_{B}{k}_{{ex}} & {p}_{A}{k}_{{ex}}\\ {p}_{B}{k}_{{ex}} & -{R}_{1B}^{0}-{p}_{A}{k}_{{ex}}\end{array}\right]\left[\begin{array}{c}\Delta {M}_{{zA}}(t)\\ \Delta {M}_{{zB}}(t)\end{array}\right]$$

In which $\Delta {M}_{{zA}}\left(t\right)={M}_{{zA}}\left(t\right)-{M}_{A}^{0}$ and $\Delta {M}_{{zB}}\left(t\right)={M}_{{zB}}\left(t\right)-{M}_{B}^{0}$. The solution of this equation is:

$$\left[\begin{array}{c}\Delta {M}_{{zA}}(t)\\ \Delta {M}_{{zB}}(t)\end{array}\right]=\left[\begin{array}{cc}{a}_{{AA}}(t) & {a}_{{AB}}(t)\\ {a}_{{BA}}(t) & {a}_{{BB}}(t)\end{array}\right]\left[\begin{array}{c}\Delta {M}_{{zA}}(0)\\ \Delta {M}_{{zB}}(0)\end{array}\right]$$

In which

$${a}_{{AA}}(t)= 	 \frac{1}{2}\bigg[\bigg(1-\frac{{R}_{1A}^{0}-{R}_{1B}^{0}+{k}_{{ex}}\left({p}_{B}-{p}_{A}\right)}{{\lambda }_{+}-{\lambda }_{-}}\bigg){{\exp }}\left(-{\lambda }_{-}t\right)\\ 	 +(1+\frac{{R}_{1A}^{0}-{R}_{1B}^{0}+{k}_{{ex}}\left({p}_{B}-{p}_{A}\right)}{{\lambda }_{+}-{\lambda }_{-}}){{\exp }}\left(-{\lambda }_{+}t\right)\bigg]$$

$${a}_{{BB}}\left(t\right)= 	 \frac{1}{2}\left[\left(1+\frac{{R}_{1A}^{0}-{R}_{1B}^{0}+{k}_{{ex}}\left({p}_{B}-{p}_{A}\right)}{{\lambda }_{+}-{\lambda }_{-}}\right){{\exp }}\left(-{\lambda }_{-}t\right)\right.\\ 	 +\left.\left(1-\frac{{R}_{1A}^{0}-{R}_{1B}^{0}+{k}_{{ex}}\left({p}_{B}-{p}_{A}\right)}{{\lambda }_{+}-{\lambda }_{-}}\right){{\exp }}\left(-{\lambda }_{+}t\right)\right]$$

$${a}_{{AB}}(t)=\frac{{k}_{{ex}}{p}_{A}}{{\lambda }_{+}-{\lambda }_{-}}\bigg[\bigg({{\exp }}\left(-{\lambda }_{-}t\right)-{{\exp }}\left({-\lambda }_{+}t\right)\bigg)\bigg]$$

$${a}_{{BA}}\left(t\right)=\frac{{k}_{{ex}}{p}_{B}}{{\lambda }_{+}-{\lambda }_{-}}\bigg[\bigg({{\exp }}\left(-{\lambda }_{-}t\right)-{{\exp }}\left({-\lambda }_{+}t\right)\bigg)\bigg]$$

and

$${\lambda }_{\pm }=\frac{1}{2}\left\{{R}_{1A}^{0}+{R}_{1B}^{0}+{k}_{{ex}}\pm \left[{\left({R}_{1A}^{0}-{R}_{1B}^{0}+{k}_{{ex}}\left({p}_{B}-{p}_{A}\right)\right)}^{2}+4{p}_{A}{p}_{B}{k}_{{ex}}^{2}\right]\right\}$$

$${p}_{A}+{p}_{B}=1$$

Despite the advantages of HSQC-detected ZZ-exchange experiments, which only require low concentration protein samples and short NMR acquisition times^75–79^, the co-existence of the folded and unfolded states in a large protein like hPFN1 leads to significant overlapping of HSQC peaks from both states and exchange cross peaks. This makes the unambiguous assignment and accurate measurement of their intensity extremely challenging. As such, in the present study, we utilized the time-consuming 3D HSQC-NOESY spectroscopy in which we have successfully identified 8 residues spanning the entire sequence with well-resolved NOE peaks resulting from the two states and exchange process (Supplementary Table 1). We subsequently measured the intensity of these NOE peaks. Together with ${R}_{1A}^{0}$ and ${R}_{1B}^{0}$ results we obtained in the separate relaxation experiments shown in Supplementary Fig. 2, ${k}_{{ex}}$, ${p}_{A}$, and ${p}_{B}$ could be derived from the above equations^43^ and presented in Supplementary Table 1.

### NMR ^15^N backbone dynamics on ps-ns time scale

^15^N backbone T1 and T1ρ relaxation times and {^1^H}-^15^N steady state NOE intensities were collected on the ^15^N-labeled WT-/C71G-hPFN1 or hSOD1 (with zinc at 1:20 or/and ATP at 1:8) at 25 °C on an Avance 800 MHz Bruker spectrometer with both an actively shielded cryoprobe and pulse field gradient units^47–50^. Relaxation time T1 was determined by collecting 7 points with delays of 10, 160, 400, 500, 640, 800, and 1000 ms using a recycle delay of 1 s, with a repeat at 400 ms. Relaxation time T1ρ was measured by collecting 8 points with delays of 1, 40, 80, 120, 160, 200, 240, and 280 ms, with a repeat at 120 ms. {^1^H}-^15^N steady-state NOEs were obtained by recording spectra with and without ^1^H presaturation, a duration of 3 s, and a relaxation delay of 6 s at 800 MHz.

### Model-free analysis

NMR relaxation data were analyzed by “Model-Free” formalism with protein dynamics software DYNAMICS^47–50^. Briefly, the relaxation of protonated heteronuclei is dominated by the dipolar interaction with the directly attached ^1^H spin and by the chemical shift anisotropy mechanism. Relaxation parameters are given by:

$${R}_{1}={d}^{2}/4[J({\omega }_{H}-{\omega }_{X})+3J({\omega }_{X})+6J({\omega }_{H}+{\omega }_{X})]+{c}^{2}J({\omega }_{X})$$

$${R}_{2}= 	 \, {d}^{2}/8[4J(0)+J({\omega }_{H}-{\omega }_{X})+3J({\omega }_{X})+6J({\omega }_{H})\\ 	 +6J({\omega }_{H}+{\omega }_{X})]+({c}^{2}/6)[4J(0)+3J({\omega }_{X})]+{R}_{ex}$$

$$NOE=1+({d}^{2}/4{R}_{1})({\gamma }_{X}/{\gamma }_{H})[6J({\omega }_{H}+{\omega }_{X})-J({\omega }_{H}-{\omega }_{X})]$$

In which,

$d={\mu }_{0}{\gamma }_{X}{\gamma }_{H}\langle {\gamma }_{XH}^{-3}\rangle /8{\pi }^{2},c={\omega }_{X}\varDelta \sigma /\sqrt{3}$, ${\mu }_{0}$ is the permeability of free space; $h$ is Planck’s constant; ${\gamma }_{X},{\gamma }_{H}$ are the gyromagnetic ratios of ^1^H and the X spin (X = ^13^C or ^15^N) respectively; ${\gamma }_{XH}$ is the X-H bond length; ${\omega }_{H}$ and ${\omega }_{X}$ are the Larmor frequencies of ^1^H and X spins, respectively; and$\varDelta \sigma$ is the chemical shift anisotropy of the X spin.

The Model-Free formalism determines the amplitudes and time scales of the intramolecular motions by modeling the spectral density function, *J*(*ω*), as

$$J(\omega )=\frac{2}{5}\left[\frac{{S}^{2}{\tau }_{m}}{1+{(\omega {\tau }_{m})}^{2}}+\frac{({{S}_{f}}^{2}-{S}^{2})\tau }{1+{(\omega \tau )}^{2}}\right]=\frac{2}{5}{{S}_{f}}^{2}\left[\frac{{{S}_{s}}^{2}\tau m}{1+{(\omega {\tau }_{m})}^{2}}+\frac{(1-{{S}_{s}}^{2})\tau }{1+{(\omega \tau )}^{2}}\right]$$

In which, $\tau ={\tau }_{s}{\tau }_{m}/({\tau }_{s}+{\tau }_{m})$, ${\tau }_{m}$ is the isotropic rotational correlation time of the molecule, ${\tau }_{s}$ is the effective correlation time for internal motions, ${S}^{2}={S}_{f}^{2}{S}_{s}^{2}$ is the square of the generalized order parameter characterizing the amplitude of the internal motions, and ${S}_{f}^{2}$ and ${S}_{s}^{2}$ are the squares of the order parameters for the internal motions on the fast and slow time scales, respectively.

In order to allow for diverse protein dynamics, several forms of the spectral density function, based on various models of the local motion, were utilized, which include the original Lipari-Szabo approach, assuming fast local motion characterized by the parameters *S*^2^ and *τ*_*loc*_; extended model-free treatment, including both fast (${{S}_{fast}}^{2},{\tau }_{fast}$) and slow (${{S}_{slow}}^{2},{\tau }_{slow}$) reorientations for the NH bond (${\tau }_{fast} < < {\tau }_{slow}\, < \, {\tau }_{c}$); and could also allow for slow, milli- to microsecond dynamics resulting in a conformational exchange contribution, *R*_*ex*_, to the linewidth^47,48,80,81^. In DYNAMICS, there are eight models for local motions and each residue is fitted with different models. Subsequently, the goodness of fit will be checked and the best-fitted model will be selected.

The relaxation data of WT-hPFN1 and the folded state of C71G-hPFN1 were analyzed with the previously published X-ray structure (pdb ID of 2PAV)^38^ by isotropic, axially-symmetric and fully anisotropic models for the overall motion and the results were tested and then compared. According to the illustration of ROTDIF, isotropic model was finally selected for both WT-hPFN1 and the folded state of C71G-hPFN1 because of the smallest Ch^2^/df value. For WT-hPFN1, τc = 7.5 ns and Dx = Dy = Dz = 1.852 E ± 07 s^−1^; for the folded state of C71G-hPFN1, τc = 7.8 ns and Dx = Dy = Dz = 2.094 E ± 07 s^−1^.

### Determination of thermodynamic stability by DSF

As ATP triggers significant noise in circular dichroism (CD) measurement, as well as the quenching effect of the intrinsic Trp fluorescence as we previously reported^19,21–23^, therefore, we were unable to use CD and fluorescence spectroscopy to determine the thermodynamic stability of C71G-hPFN1 in the presence of ATP and PPP by performing chemical and thermal unfolding as usually conducted^37^. Here we thus measured the thermodynamic stability of the WT- and C71G-hPFN1 by differential scanning fluorimetric (DSF) method^58^, as we previously used to characterize the effect of ATP on other folded proteins^21–23^. DSF was used to determine the thermodynamic stability of WT-hPFN1 and C71G-hPFN1 at protein concentrations of 10 μM in 1 mM sodium phosphate buffer containing 2 mM DTT (pH 6.0) in the presence of ATP at different concentrations. DSF experiments were performed using the CFX384 Touch™ Real- Time PCR Detection System from BIO-RAD, following the SYBR green melting protocol to obtain Tm value. Briefly, in a single well of a 384-well PCR plate, a 10-μL reaction solution was placed, which contains the proteins at 10 μM, ATP at different concentrations, and 10× SYPRO Orange. Plates were sealed with a quantitative PCR adhesive optical seal sheet (Microseal ‘B’ Adhesive Sealing Films, BIO-RAD) and then spun at 1000 rpm for 1 min to remove bubbles. The program in Real-Time PCR instrument was set to SYBR green and ran the temperature scan from 25 °C to 95 °C with the increment of 1 °C/min. Upon completion, the obtained thermal unfolding curves were displayed as the first derivatives (dF/dT) by the reverse transcriptase PCR software Bio-Rad CFX Manager 3.0.

### Reporting summary

Further information on research design is available in the Nature Portfolio Reporting Summary linked to this article.

### Supplementary information

SupportingInformation

Description of Additional Supplementary Files

Supplementary Data 1

Reporting Summary

## Data Availability

The authors declare that the main data supporting the findings of this study are available within the article and its Supplementary Information files. The raw data for Figs. 2c and 2c, Figs. 5c and 5d, Fig. 8a are presented in Supplementary Data 1.
