# Supplementary material for: ATP induces folding of ALS-causing C71G-hPFN1 and nascent hSOD1
Source: Commun Chem. 2023 Sep 5;6:186. doi: 10.1038/s42004-023-00997-0 (PMC10480188; doi:10.1038/s42004-023-00997-0)
Supplement: Supplementary file 1 — SupportingInformation [file 42004_2023_997_MOESM1_ESM.pdf]

Supporting Information

ATP induces folding of ALS-causing C71G-hPFN1 and nascent hSOD1

Jian Kang, Liangzhong Lim and Jianxing Song*

Department of Biological Sciences, Faculty of Science, National University of Singapore; 10 Kent Ridge Crescent, Singapore 119260;

This PDF file contains:

Supplementary Table 1
Supplementary Figure 1 to 20

Supplementary Table

Supplementary Table 1. Populations and kex of C71G-hPFN1.

Residue	Population (%) (Unfolded)	Population (%) (Folded)	kex (Hz)
C17	46.2	53.8	11.7
V31	47.4	52.6	11.8
T65	48.1	51.9	11.5
V103	42.4	57.6	11.4
G115	45.1	54.9	11.5
V119	43.1	56.9	11.8
M131	43.0	57.0	11.7
L135	42.9	57.1	11.9
Average	44.8 ± 2.2	55.2 ± 1.9	11.7 ± 0.2

Supplementary Figures

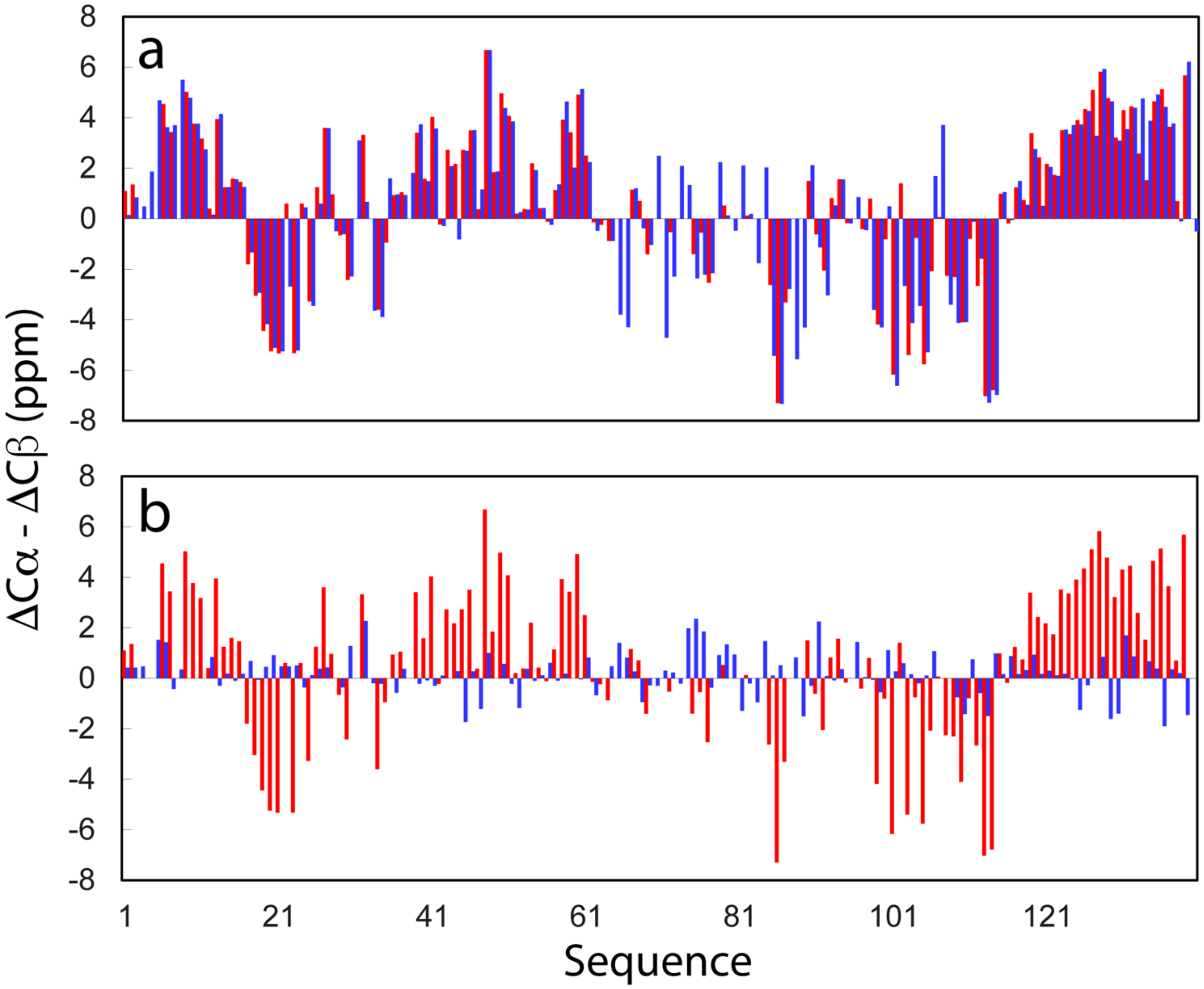

Supplementary Figure 1. NMR conformations of WT-hPFN1, folded and unfolded states of C71G-hPFN1.

(a) Residue specific (ΔCα-ΔCβ) chemical shifts of WT-hPFN1 (blue) and folded state of C71G-hPFN1 (red). (b) Residue specific (ΔCα-ΔCβ) chemical shifts of folded state (red) and unfolded state of C71G-hPFN1 (blue).

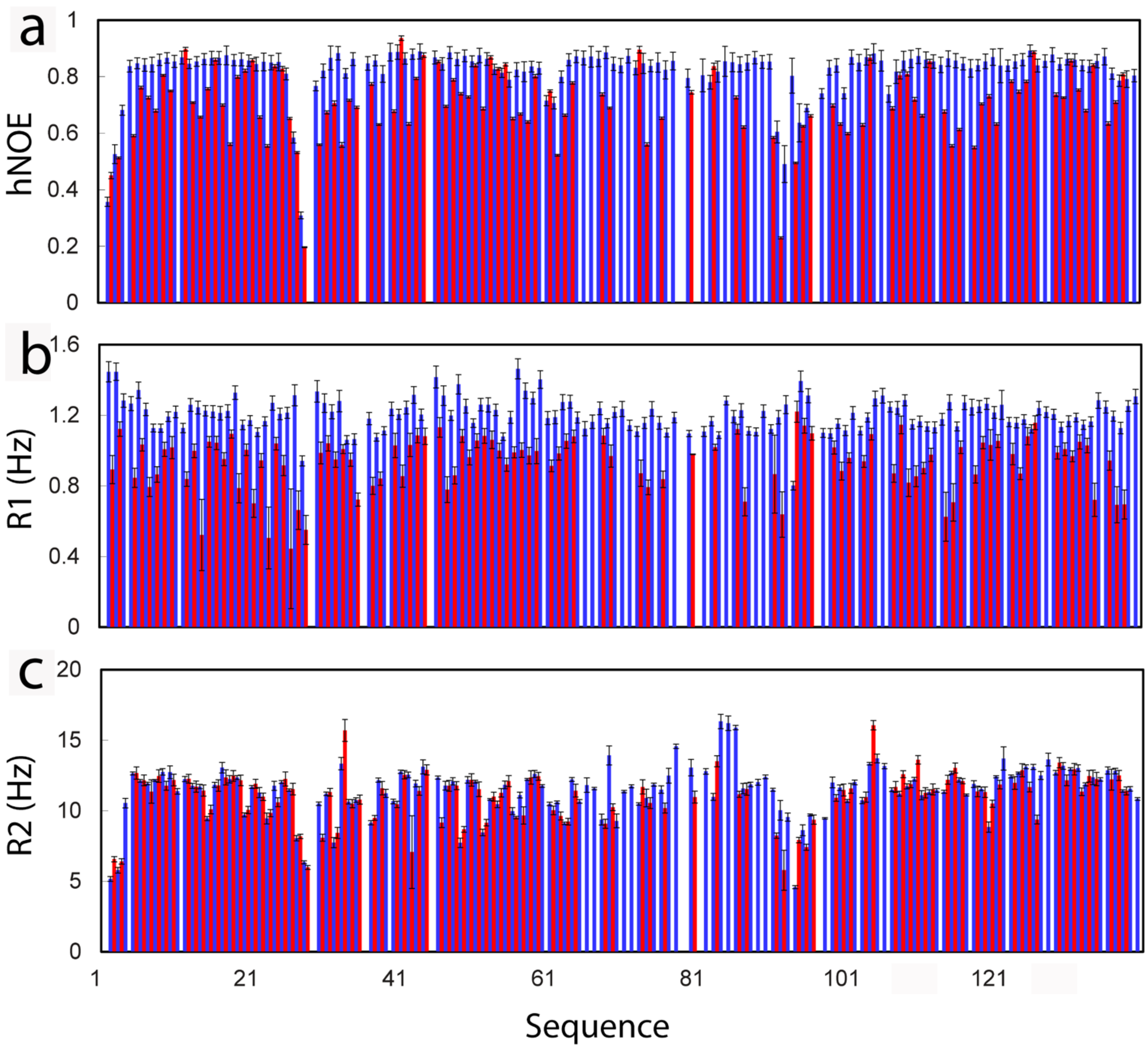

Supplementary Figure 2. ^{15}N NMR backbone relaxation data of WT-PFN1 and C71G-hPFN1. ^{15}N NMR backbone relaxation data of WT-hPFN1 (blue) and folded state of C71G-hPFN1 (red), as measured at 800 MHz. (a) $\{^{1}H\}$-^{15}N steady-state NOE intensities. (b) R1. (c) R2. The error bars were generated during the fitting processes.

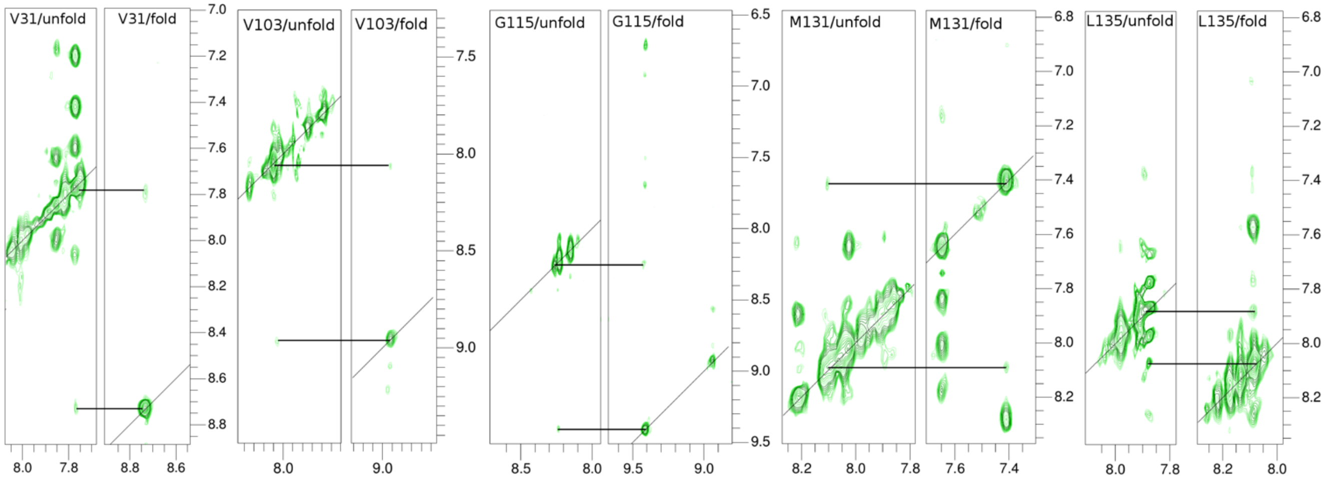

Supplementary Figure 3. Conformational exchange of folded and unfolded states of C71G-hPFN1. Strips of ^{15}N-edited three-dimensional HSQC-NOESY spectrum of C71G-hPFN1 showing the cross peaks which result from the conformational exchange of amide protons of representative residues between the folded and unfolded states.

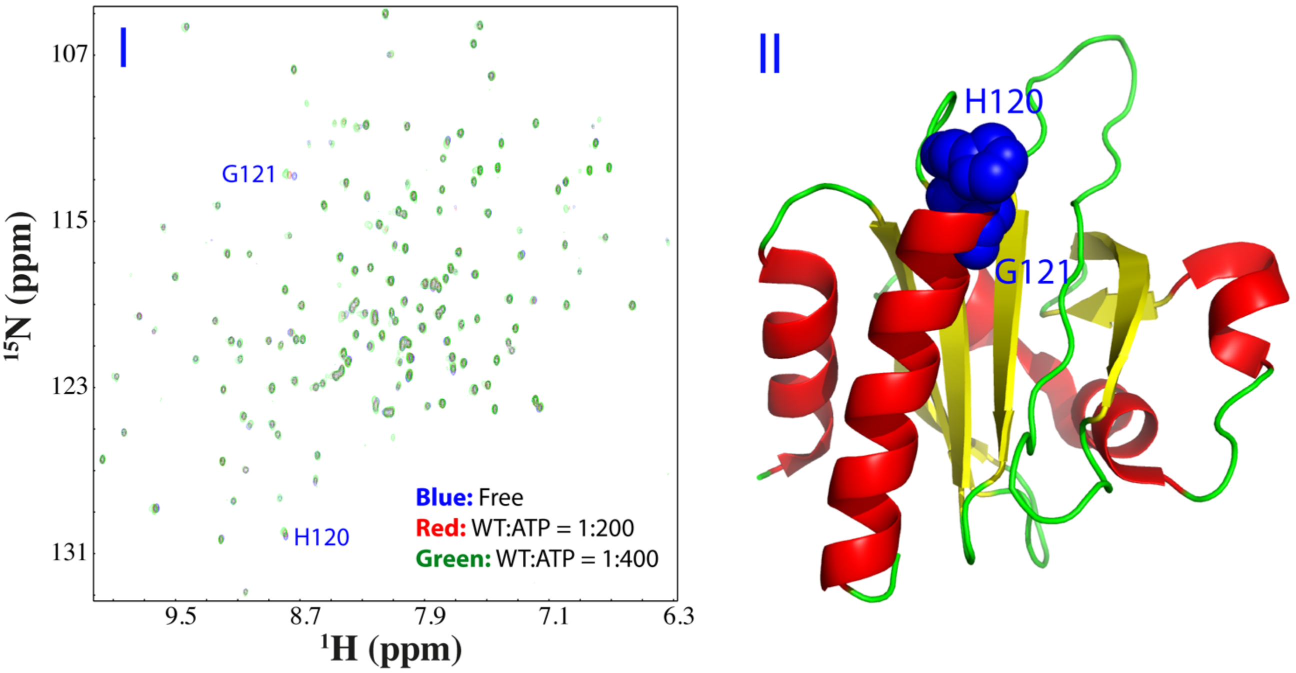

Supplementary Figure 4. ATP has no binding pocket on WT-hPFN1.
(I) ^{1}H-^{15}N HSQC spectra of WT-hPFN1 in the absence and in the presence of ATP at different ratios. Two residues (His120 and Gly121) with shifted HSQC peaks are labeled. (II) Crystal structure of the human profilin-1 with two residues His120 and Gly121 displayed in blue spheres.

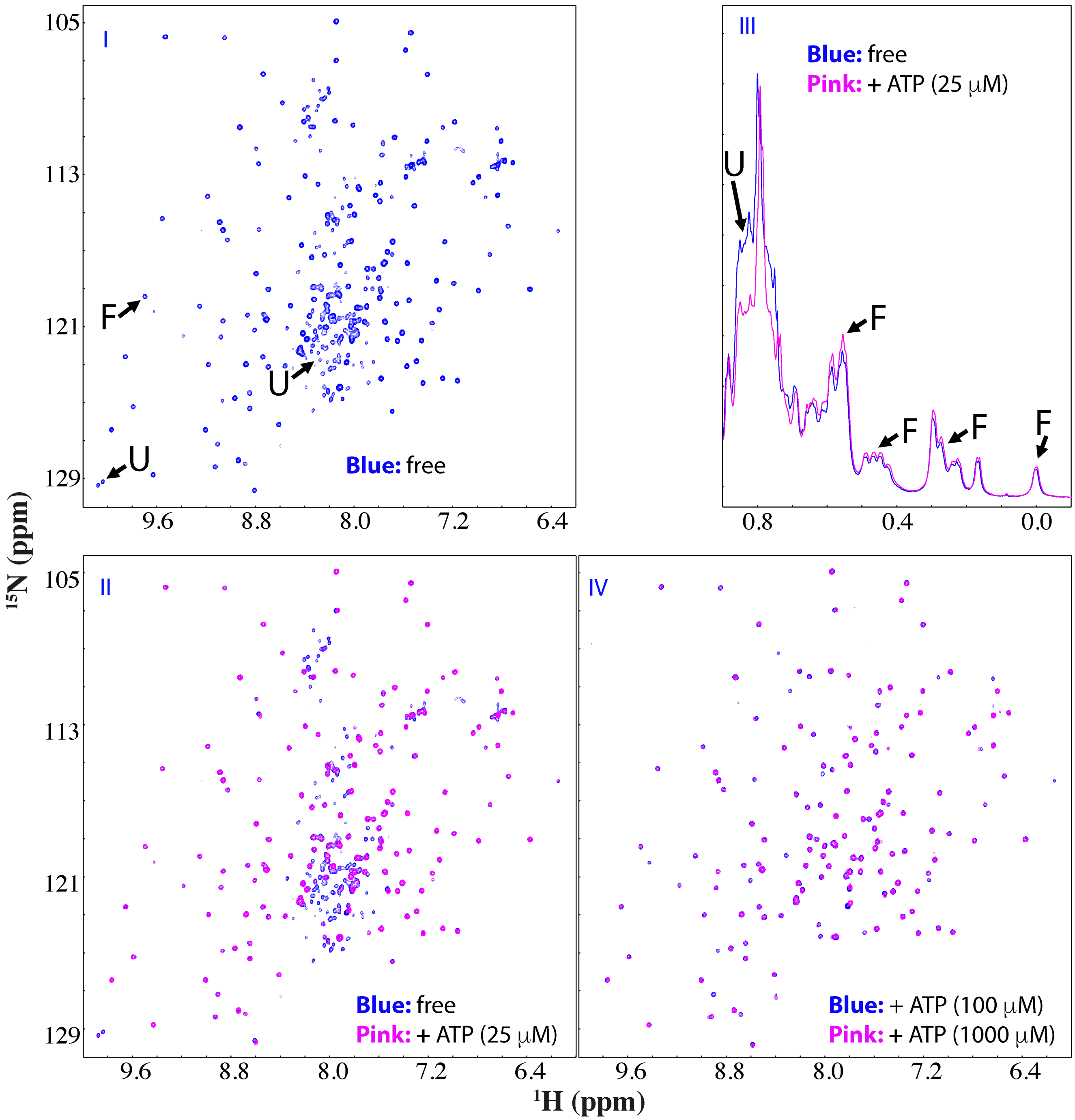

Supplementary Figure 5. ATP shifts the conformational equilibrium to favor the folded state. (I) Two-dimensional HSQC spectrum of ^{15}N-labeled C71G-hPFN1 at a concentration of 50 μM. (II) Superimposition of HSQC spectra of ^{15}N-labeled C71G-hPFN1 in the absence (blue) and in the presence of ATP (pink) at a molar ratio of 1:0.5. (III) Up-field 1D NMR spectra of C71G-hPFN1 in the presence of ATP at different ratios. (IV) Superimposition of HSQC spectra of ^{15}N-labeled C71G-hPFN1 in the presence of ATP at a molar ratio of 1:2 (blue) and 1:20 (pink). Some characteristic NMR signals of the folded (F) and unfolded (U) states were indicated by arrows.

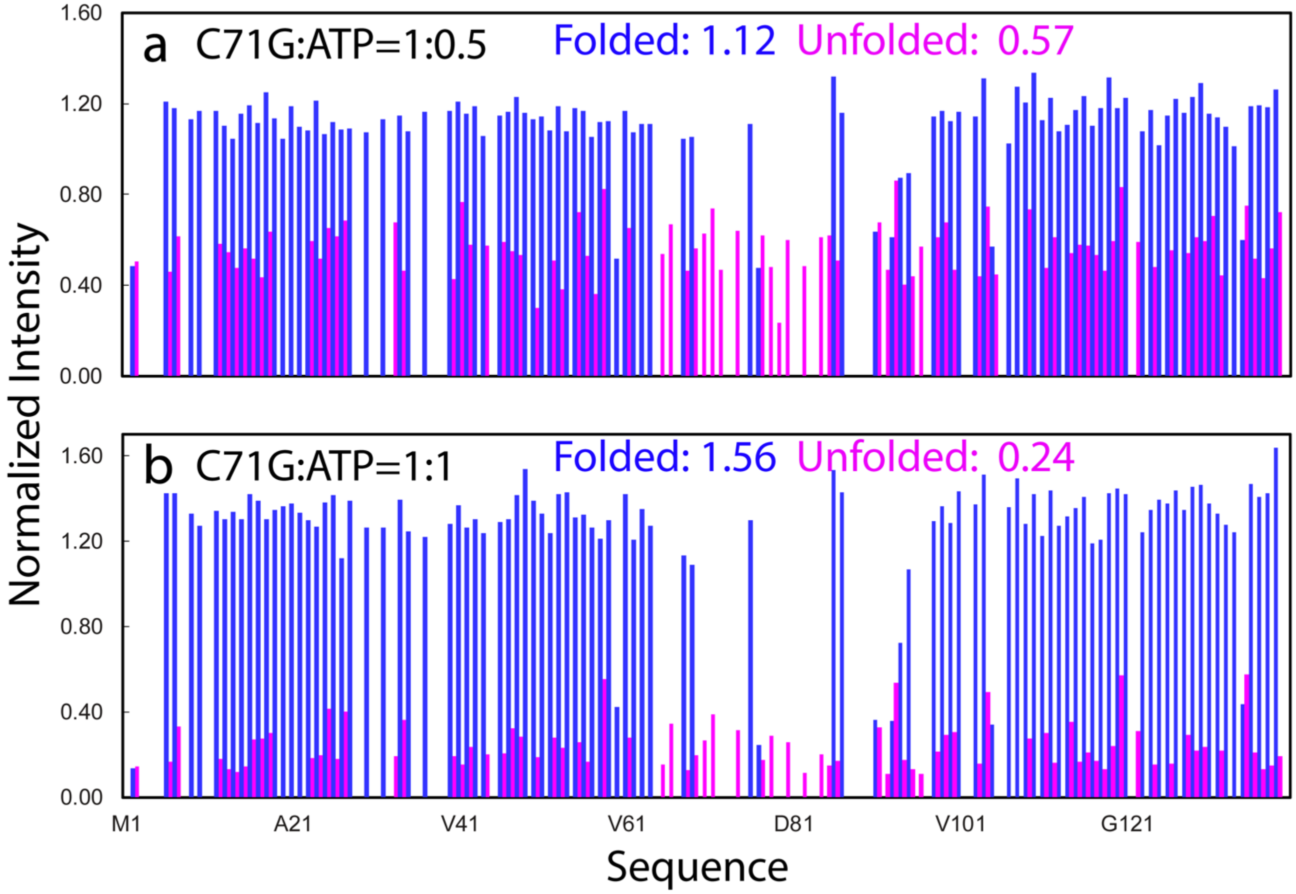

Supplementary Figure 6. ATP-induced change of HSQC peak intensity of folded and unfolded states of C71G-hPFN1.

Normalized HSQC peak intensity of folded (blue) and unfolded (pink) states of C71G-hPFN1 in the presence of ATP at molar ratios of 1:0.5 (a) and 1:1 (b), as divided by those of their corresponding peak intensity of C71G-hPFN1 in the free state. The average values are presented.

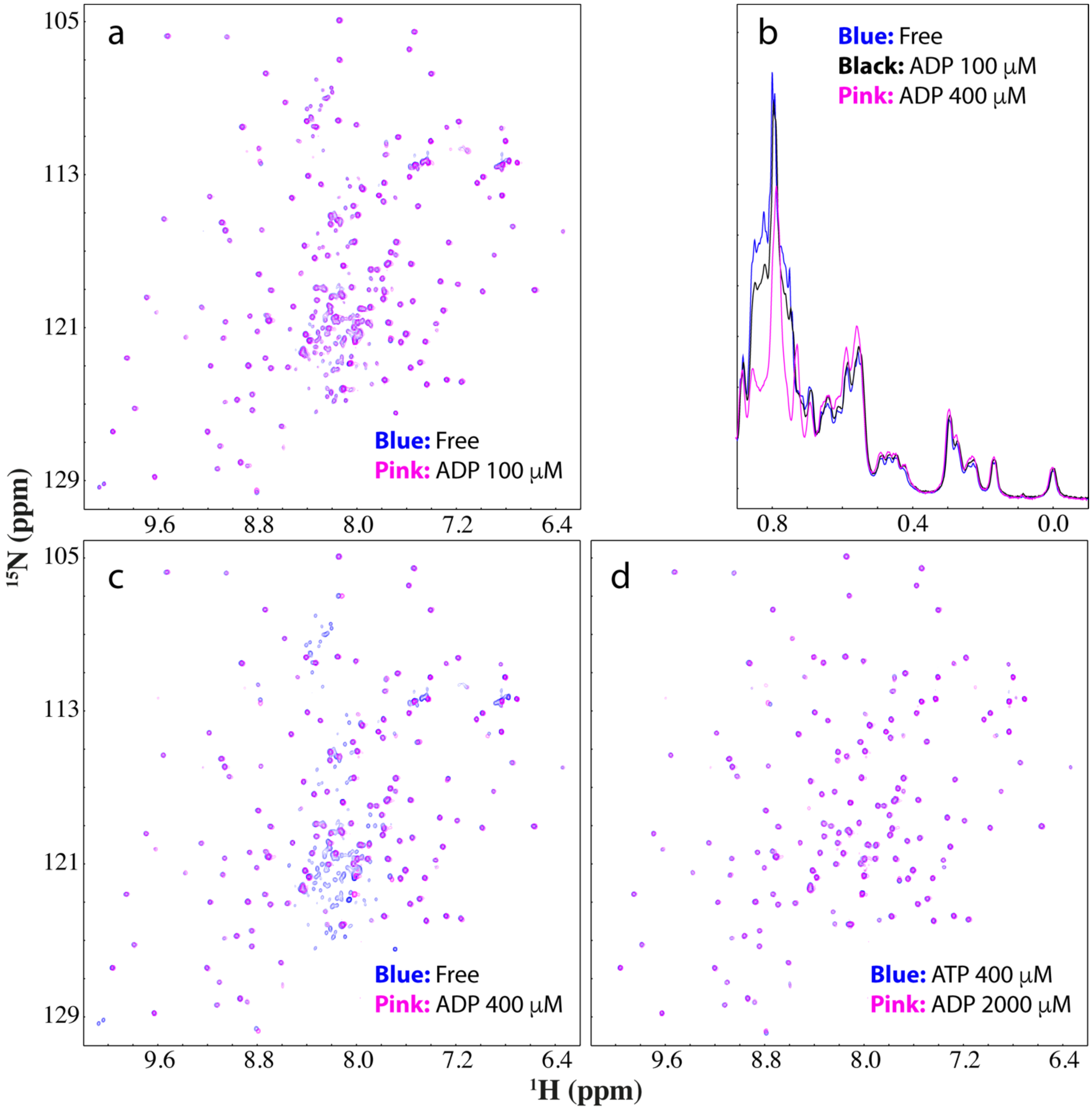

Supplementary Figure 7. ADP can also shift the conformational equilibrium to favor the folded state.

(a) Superimposition of HSQC spectra of ^{15}N-labeled C71G-hPFN1 at a concentration of 50 μM in the absence (blue) and in the presence of ADP (pink) at a molar ratio of 1:2. (b) Up-field 1D NMR spectra of C71G-hPFN1 in the presence of ADP at different ratios. (c) Superimposition of HSQC spectra of ^{15}N-labeled C71G-hPFN1 in the absence (blue) and in the presence of ADP (pink) at a molar ratio of 1:8. (d) Superimposition of HSQC spectra of ^{15}N-labeled C71G-hPFN1 in the presence of ATP at a molar ratio of 1:8 (blue) and ADP at 1:40 (pink).

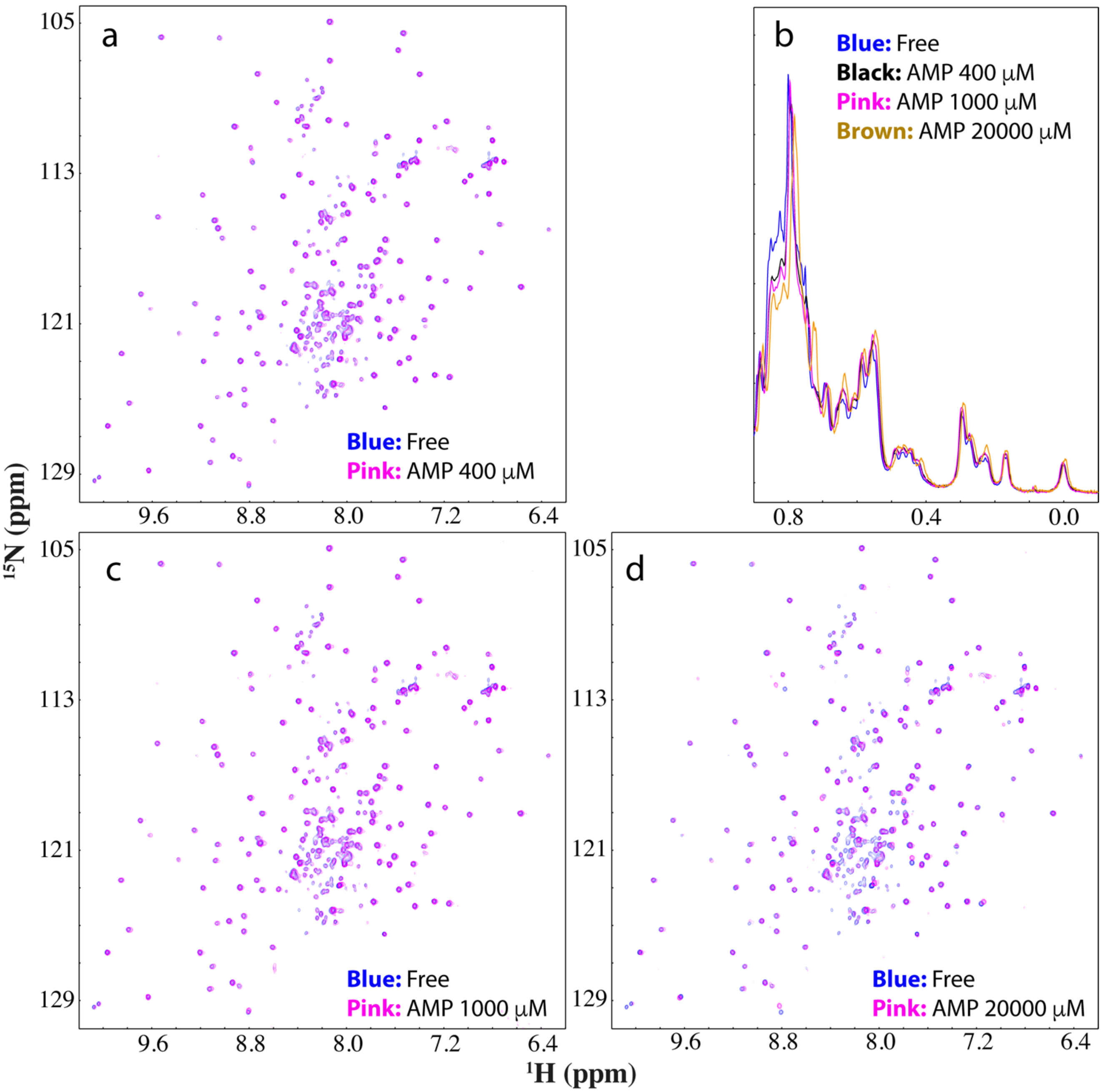

Supplementary Figure 8. AMP has no capacity in completely shifting the conformational equilibrium.

(a) Superimposition of HSQC spectra of ^{15}N-labeled C71G-hPFN1 at a concentration of 50 μM in the absence (blue) and in the presence of AMP (pink) at a molar ratio of 1:8. (b) Up-field 1D NMR spectra of C71G-hPFN1 in the presence of AMP at different ratios. (c) Superimposition of HSQC spectra of ^{15}N-labeled C71G-hPFN1 in the absence (blue) and in the presence of AMP (pink) at a molar ratio of 1:20. (d) Superimposition of HSQC spectra of ^{15}N-labeled C71G-hPFN1 in the absence (blue) and in the presence of AMP (pink) at a molar ratio of 1:400.

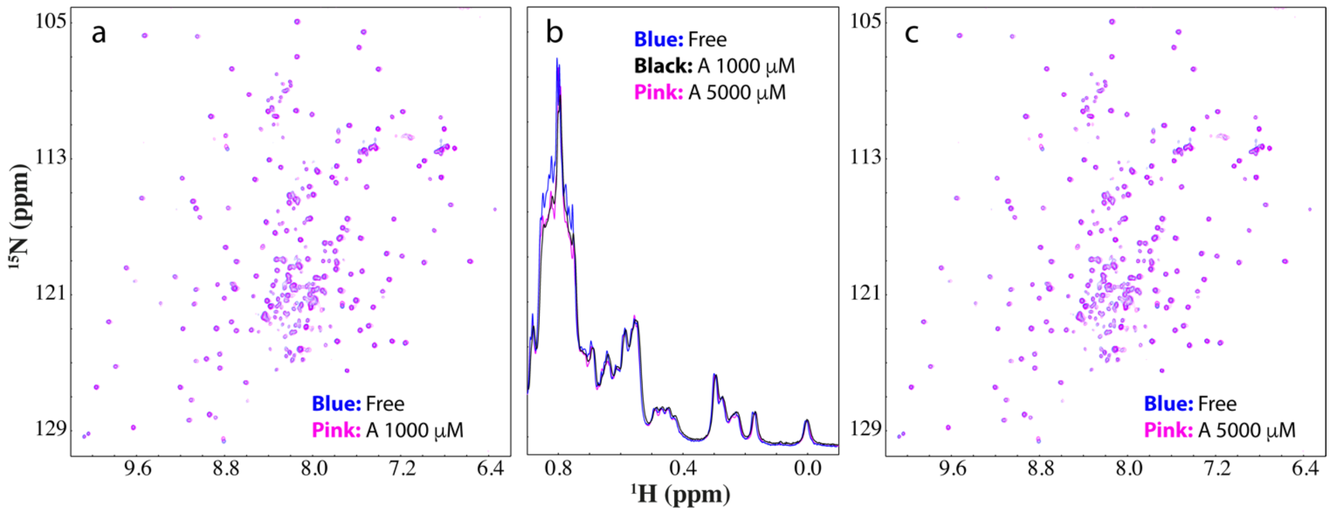

Supplementary Figure 9. Adenosine has no significant capacity in shifting the conformational equilibrium.

(a) Superimposition of HSQC spectra of ^{15}N-labeled C71G-hPFN1 at a concentration of 50 μM in the absence (blue) and in the presence of Adenosine (pink) at a molar ratio of 1:20. (b) Up-field 1D NMR spectra of C71G-hPFN1 in the presence of Adenosine at different ratios. (c) Superimposition of HSQC spectra of ^{15}N-labeled C71G-hPFN1 in the absence (blue) and in the presence of Adenosine (pink) at a molar ratio of 1:100.

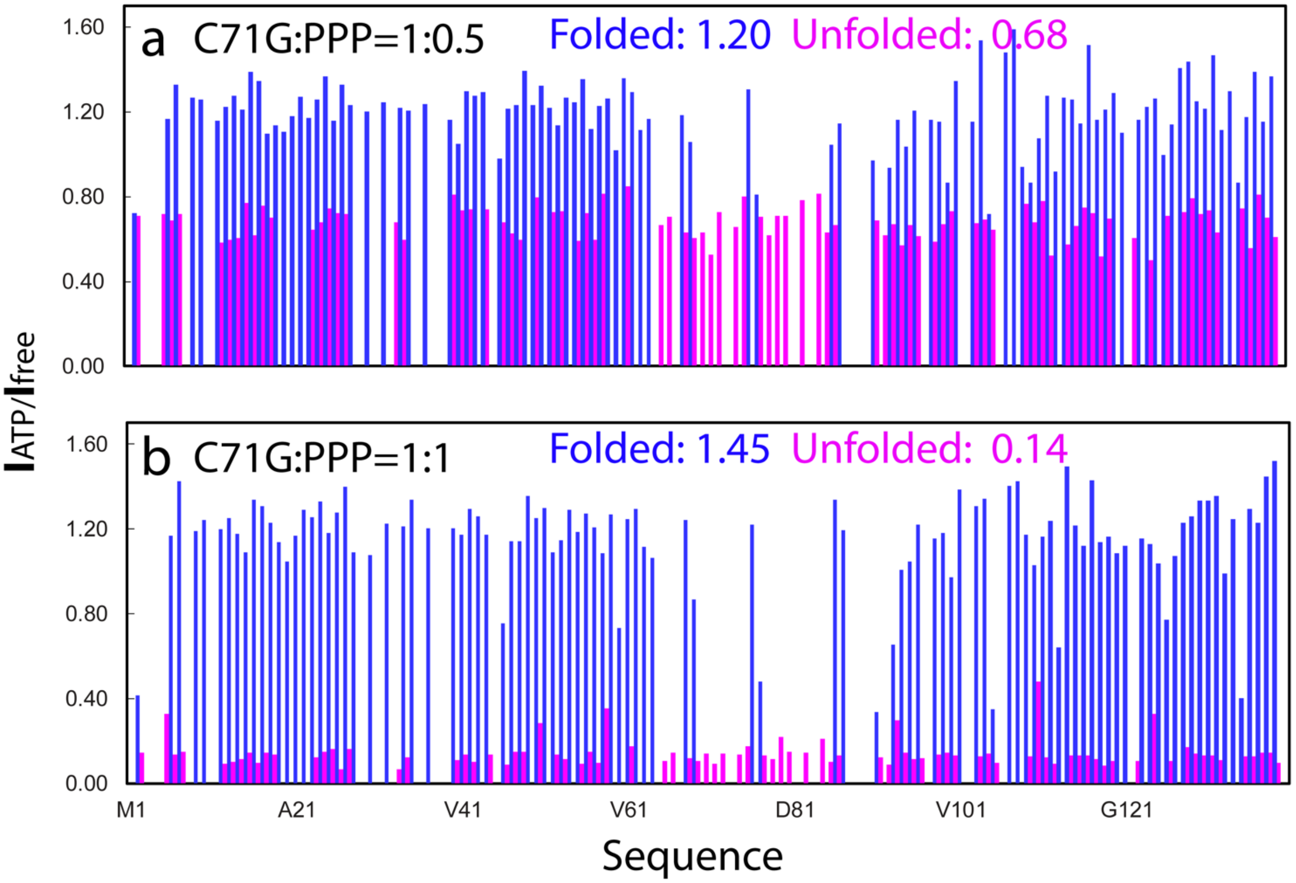

Supplementary Figure 10. PPP-induced change of HSQC peak intensity of folded and unfolded states of C71G-hPFN1.

Normalized HSQC peak intensity of the folded (blue) and unfolded (pink) states the ^{15}N-labeled C71G-hPFN1 in the presence of PPP at molar ratios of 1:0.5 (A) and 1:1 (B), as divided by those of their corresponding peak intensity of C71G-hPFN1 in the free state. The average values are presented.

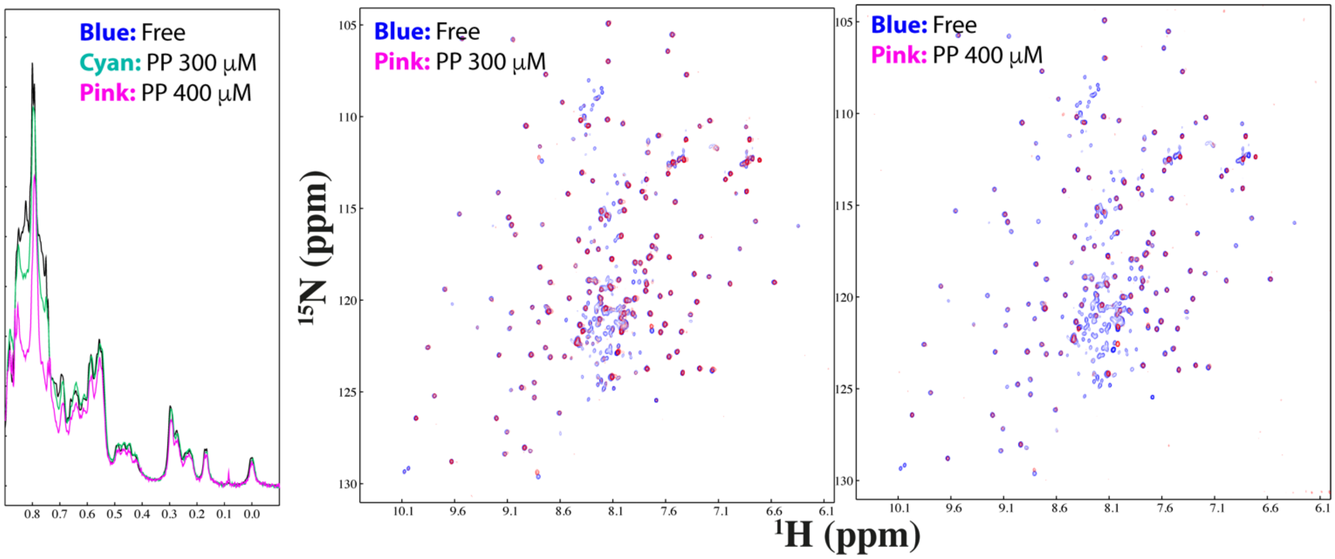

Supplementary Figure 11. Sodium pyrophosphate has capacity in shifting the conformational equilibrium.

Superimposition of up-field 1D and HSQC spectra of ^{15}N-labeled C71G-hPFN1 at a concentration of 50 μM in the absence and in the presence of sodium pyrophosphate at different concentrations.

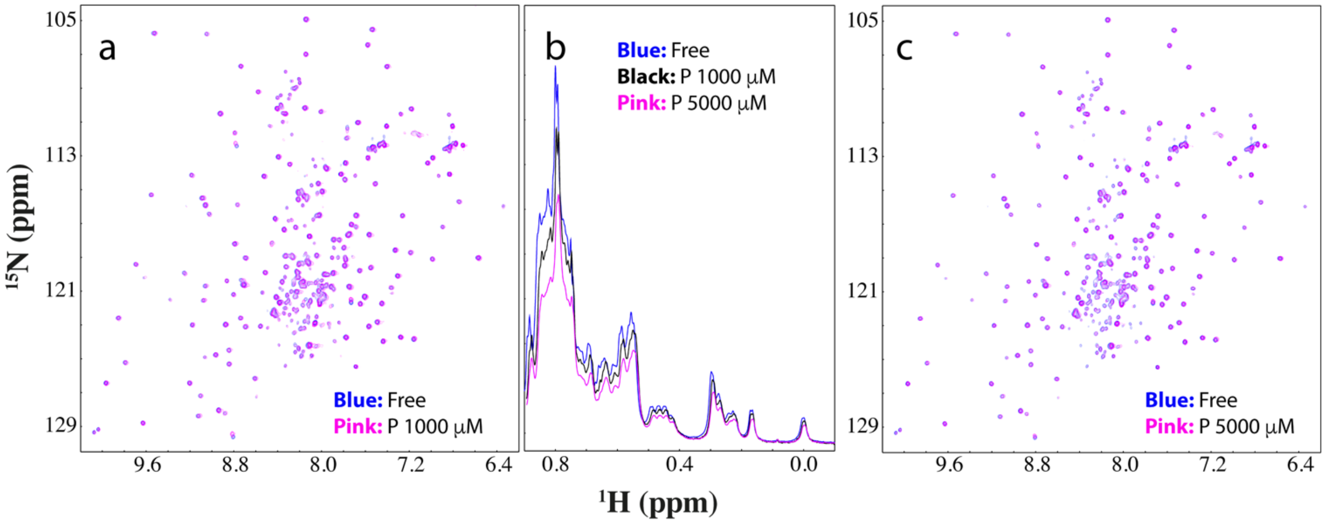

Supplementary Figure 12. Sodium phosphate has no capacity in shifting the conformational equilibrium.

(a) Superimposition of HSQC spectra of ^{15}N-labeled C71G-hPFN1 at a concentration of 50 μM in the absence (blue) and with the additional addition of sodium phosphate (pink) at a molar ratio of 1:20. (b) Up-field 1D NMR spectra of C71G-hPFN1 in the presence of sodium phosphate at different ratios. (c) Superimposition of HSQC spectra of ^{15}N-labeled C71G-hPFN1 in the absence (blue) and with the additional addition of sodium phosphate (pink) at a molar ratio of 1:100.

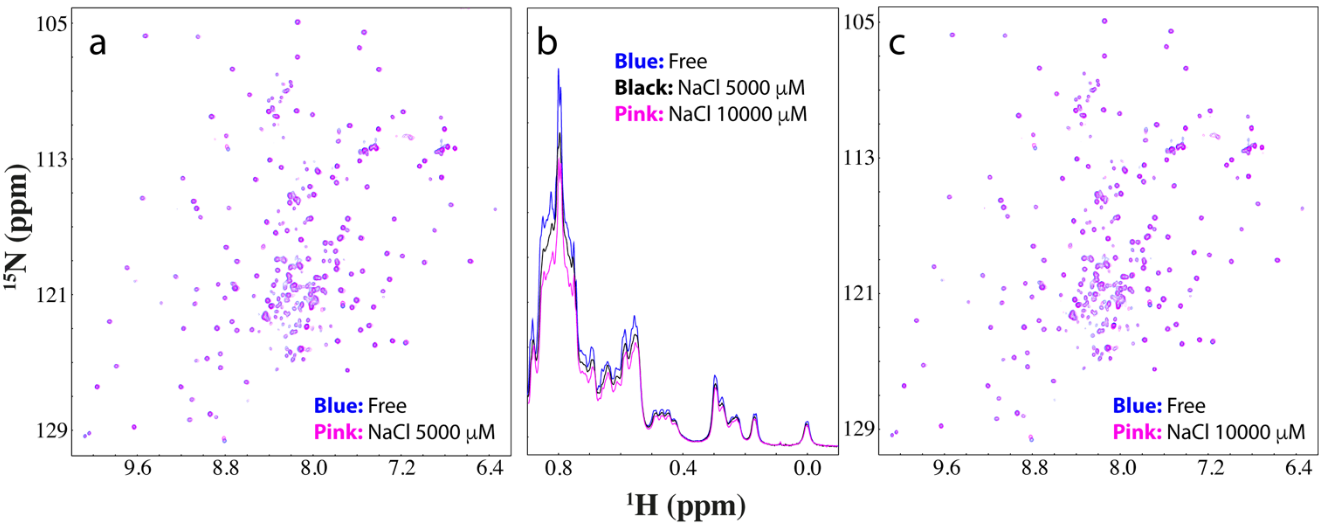

Supplementary Figure 13. Sodium chloride has no capacity in shifting the conformational equilibrium.

(a) Superimposition of HSQC spectra of ^{15}N-labeled C71G-hPFN1 at a concentration of 50 μM in the absence (blue) and in the presence of NaCl (pink) at a molar ratio of 1:100. (b) Up-field 1D NMR spectra of C71G-hPFN1 in the presence of NaCl at different ratios. (c) Superimposition of HSQC spectra of ^{15}N-labeled C71G-hPFN1 in the absence (blue) and in the presence of NaCl (pink) at a molar ratio of 1:200.

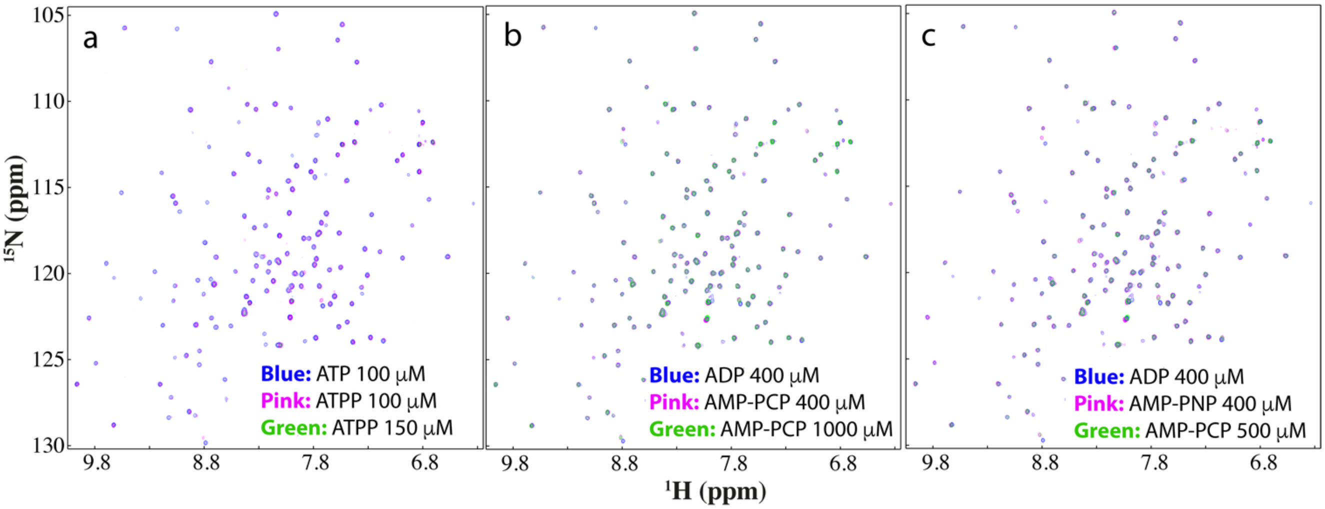

Supplementary Figure 14. The capacity of ATPP, AMP-PCP and AMP-PNP in shifting the conformational equilibrium.

(a) Superimposition of HSQC spectra of ^{15}N-labeled C71G-hPFN1 at a concentration of 50 μM in the presence of ATP at 100 μM (blue), ATPP at 100 μM (pink) and 150 μM (green). (b) Superimposition of HSQC spectra of ^{15}N-labeled C71G-hPFN1 at a concentration of 50 μM in the presence of ADP at 400 μM (blue), AMP-PCP at 400 μM (pink) and 1000 μM (green). (c) Superimposition of HSQC spectra of ^{15}N-labeled C71G-hPFN1 at a concentration of 50 μM in the presence of ADP at 400 μM (blue), AMP-PNP at 400 μM (pink) and 500 μM (green).

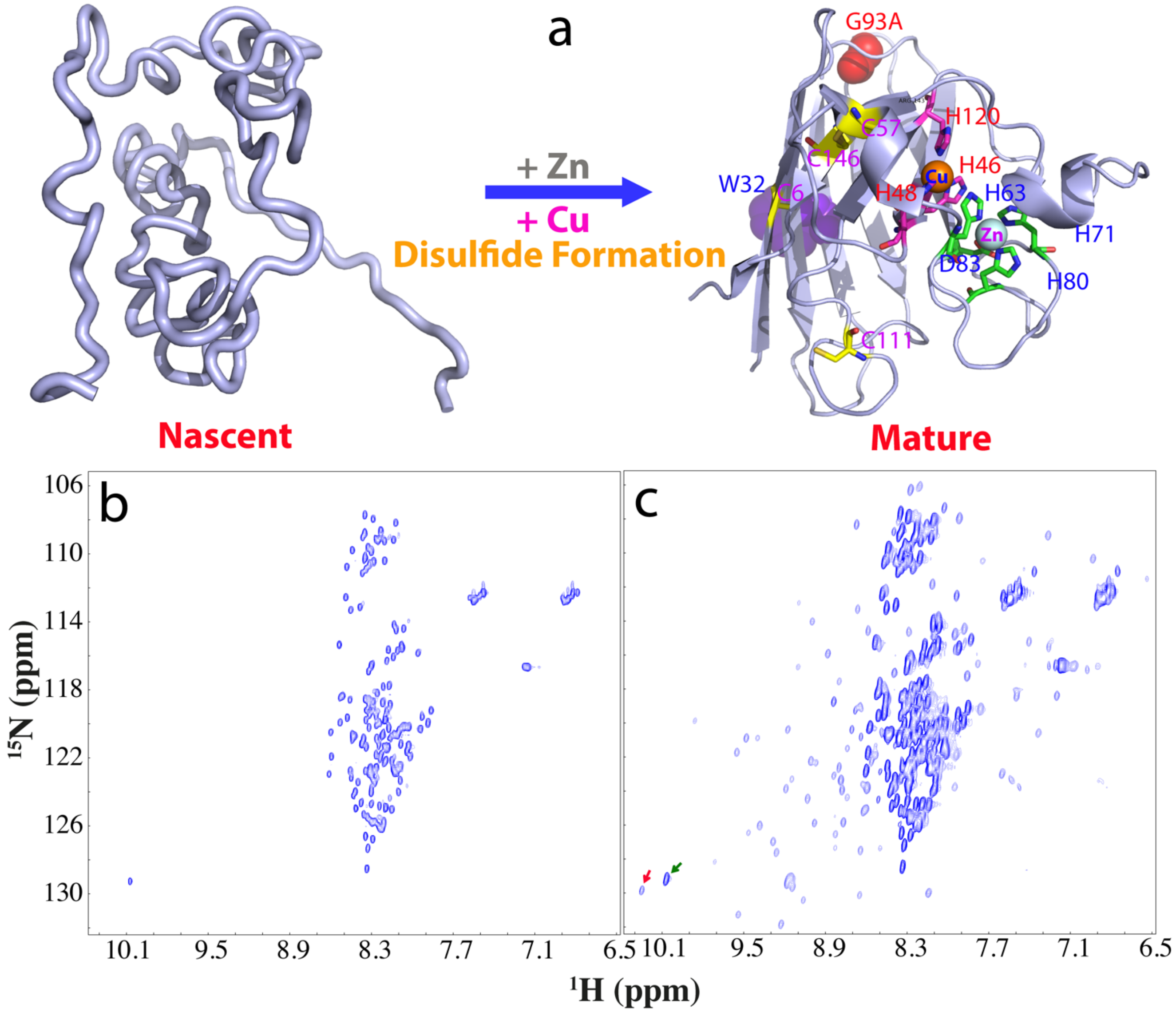

Supplementary Figure 15. Zn2+ specifically binds and initiates folding of nascent hSOD1.
(a) Schematic representation of the maturation process of hSOD1 from the unfolded nascent state to mature subunit. Mature hSOD1 is represented by the monomeric subunit of the dimeric crystal structure of hSOD1 (PDB ID of 2C9V). Residues critical for binding metal cofactors are labeled. (b) Two-dimensional 1H-15N NMR HSQC spectrum of nascent hSOD1 of WT sequence without metalation and disulfide bridge. (c) HSQC spectrum of nascent hSOD1 in the presence of zinc at a ratio of 1:20 (hSOD1:zinc). Green arrow is used for indicating HSQC peak of the Trp32 ring proton resulting from the unfolded state while red arrow for that from the folded hSOD1.

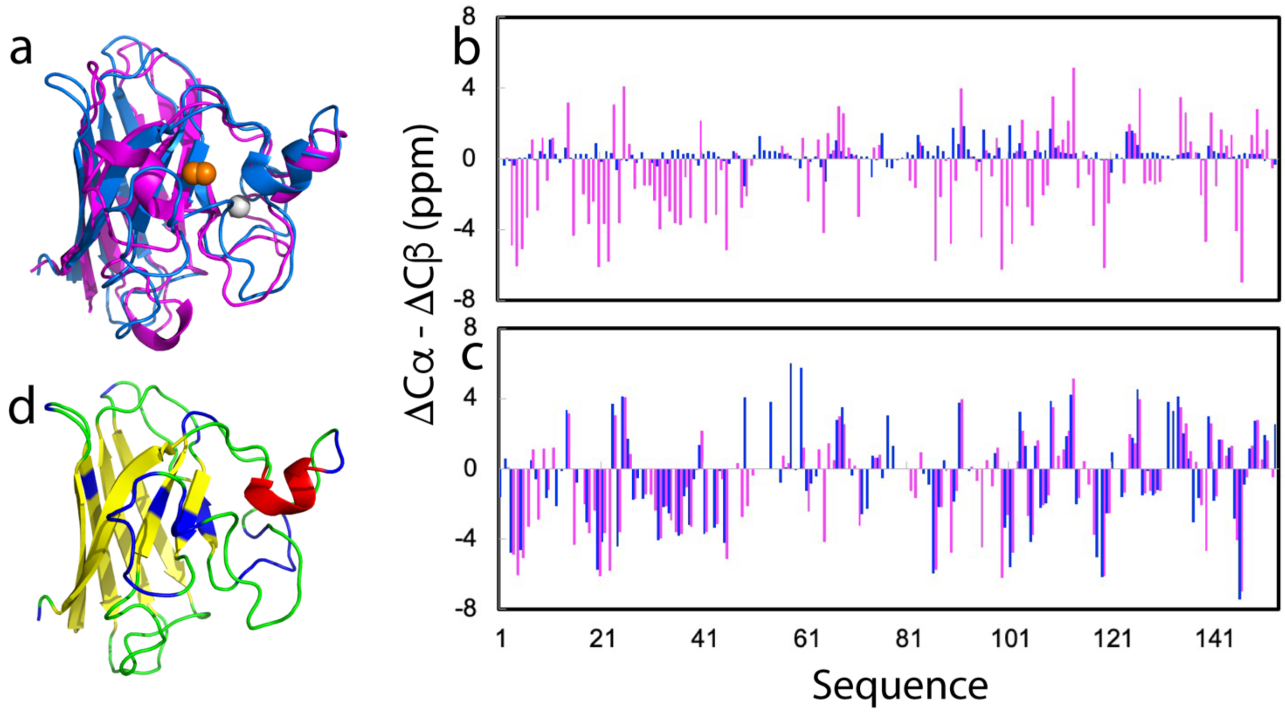

Supplementary Figure 16. NMR characterization of different forms of hSOD1.
(a) Superimposition of NMR structure of the super-stable pseudo-WT hSOD1 C6A/C111S (PDB ID of 2AF2) without the disulfide bridge and copper (blue) and crystal structure (PDB ID of 2C9V) of the mature hSOD1 (purple). Zinc ion is in grey sphere and copper in orange sphere. (b) Residue specific (ΔCα–ΔCβ) chemical shifts of nascent hSOD1 (blue) and the Zn^{2+}-induced hSOD1 (purple), (c) Residue specific (ΔCα–ΔCβ) chemical shifts of the Zn^{2+}-induced hSOD1 (purple), and those of C6A/C111S (blue) (BMRB Entry of 6821) (Ref. 12). (d) NMR structure of C6A/C111S with the unassigned residues of the Zn^{2+}-induced folded state colored in blue due to missing or overlapping resonances.

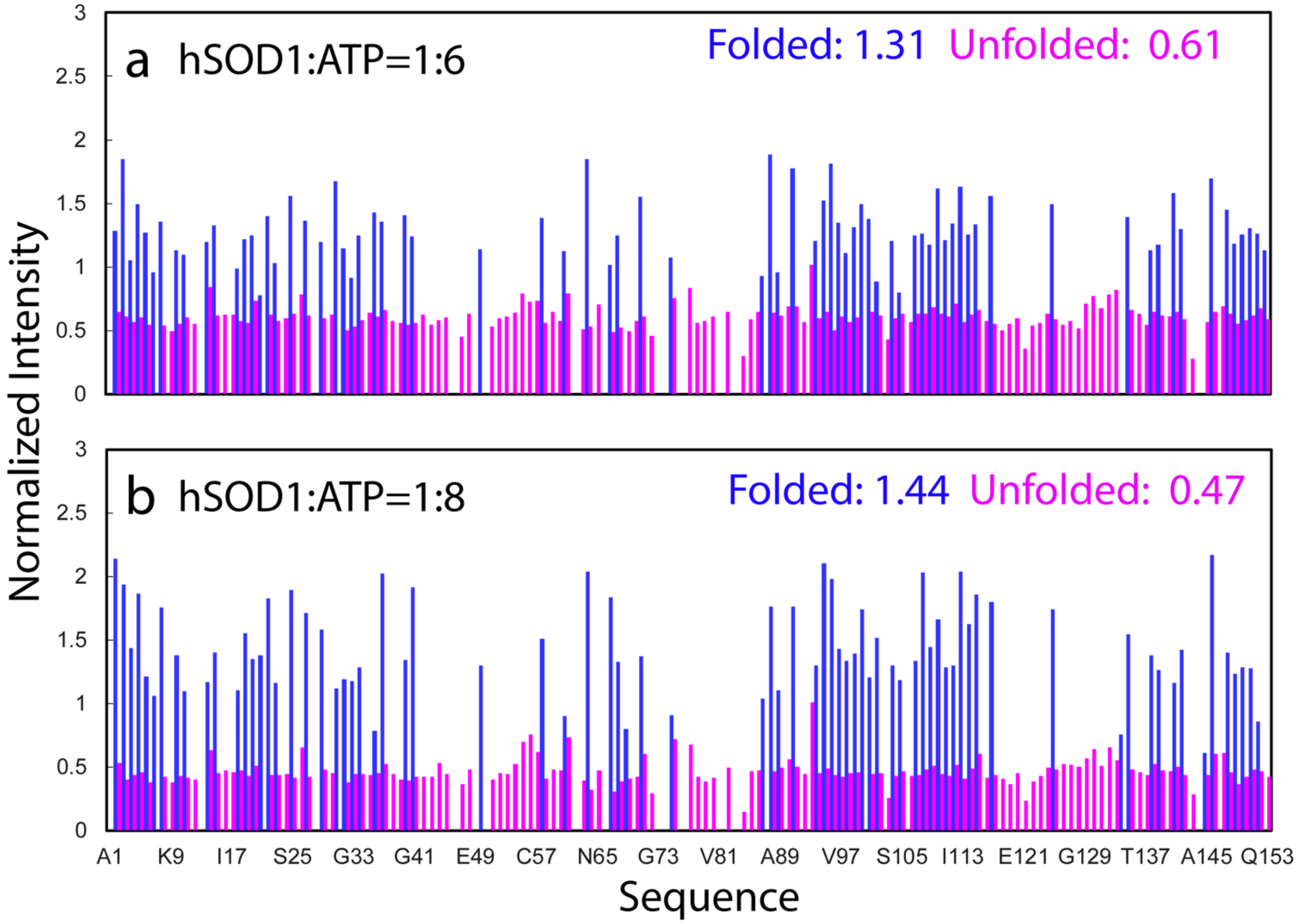

Supplementary Figure 17. ATP-induced folding of nascent hSOD1.

Normalized HSQC peak intensity of the folded (blue) and unfolded (pink) states of the ^{15}N-labeled hSOD1 in the presence of ATP at molar ratios of 1:6 (a) and 1:8 (b), as divided by those of their corresponding peak intensity of hSOD1 in the presence of ATP at 1:4. The average values are presented.

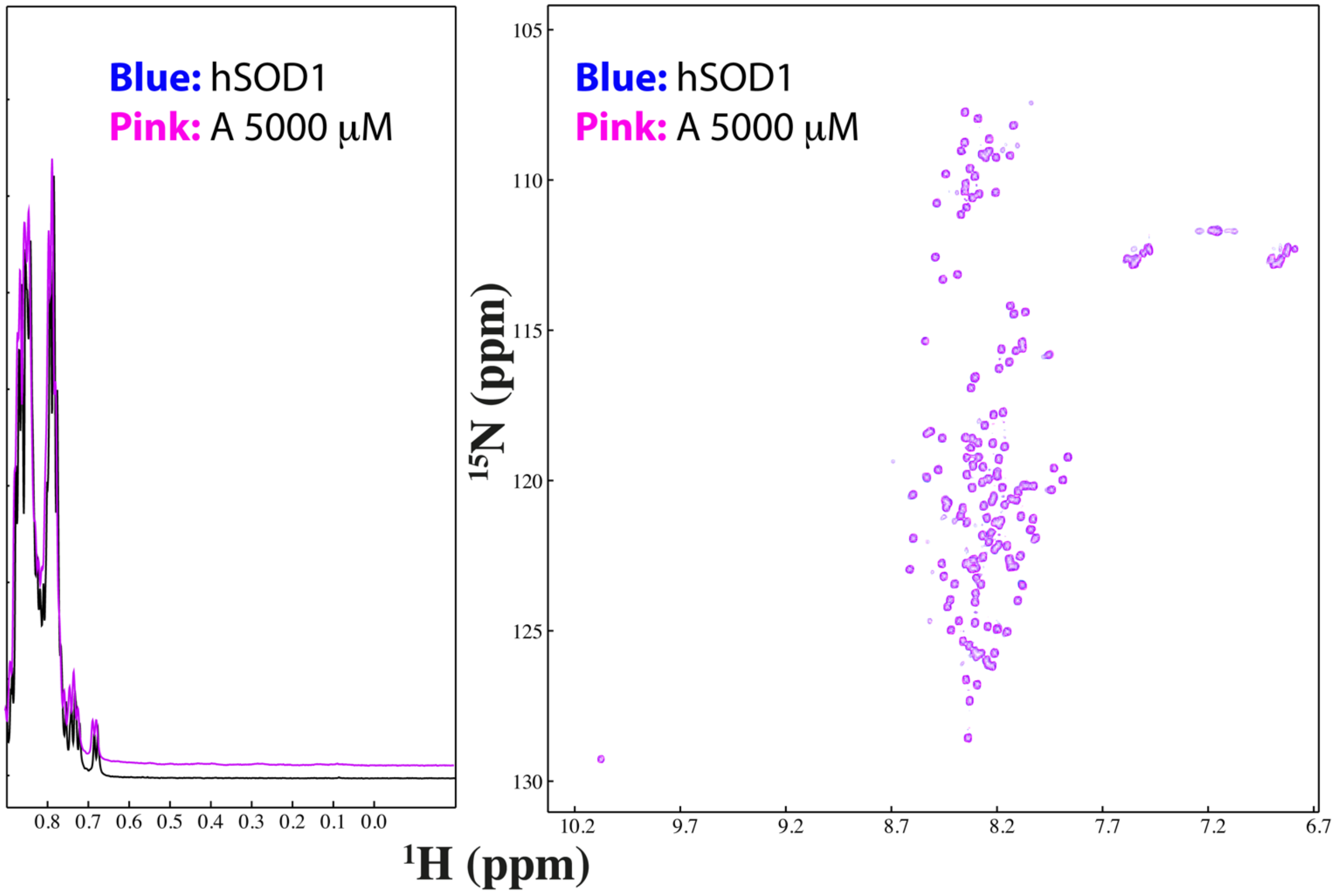

Supplementary Figure 18. Adenosine failed to induce folding of nascent hSOD1.
Up-field NMR 1D and HSQC spectra of ^{15}N-labeled hSOD1 in the absence (blue) and in the presence of Adenosine at 1:100 (purple).

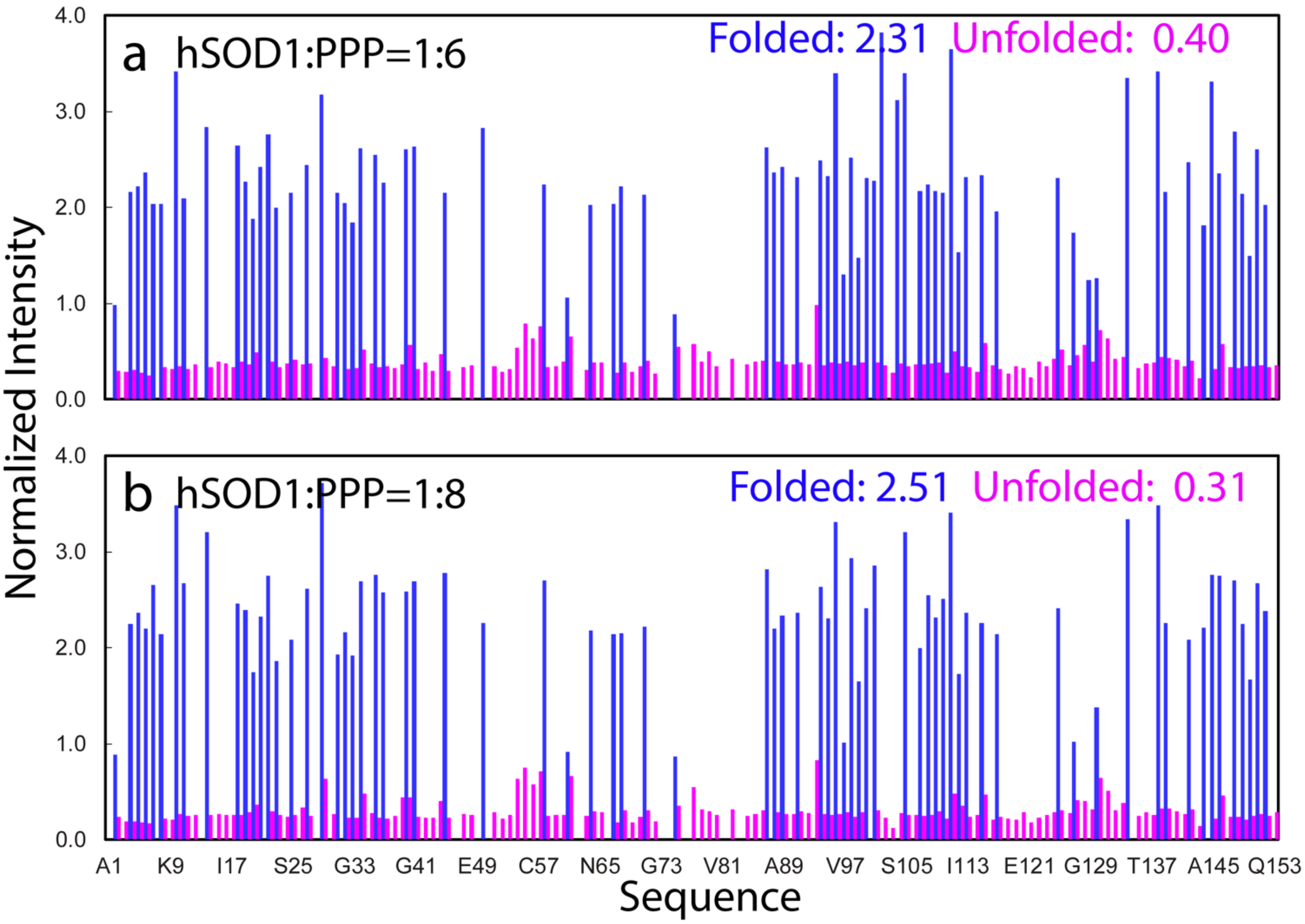

Supplementary Figure 19. PPP induces folding of nascent hSOD1.
Normalized HSQC peak intensity of the folded (blue) and unfolded (pink) states of the ^{15}N-labeled hSOD1 in the presence of PPP at molar ratios of 1:6 (a) and 1:8 (b), as divided by those of their corresponding peak intensity of hSOD1 in the presence of PPP at 1:4. The average values are presented.

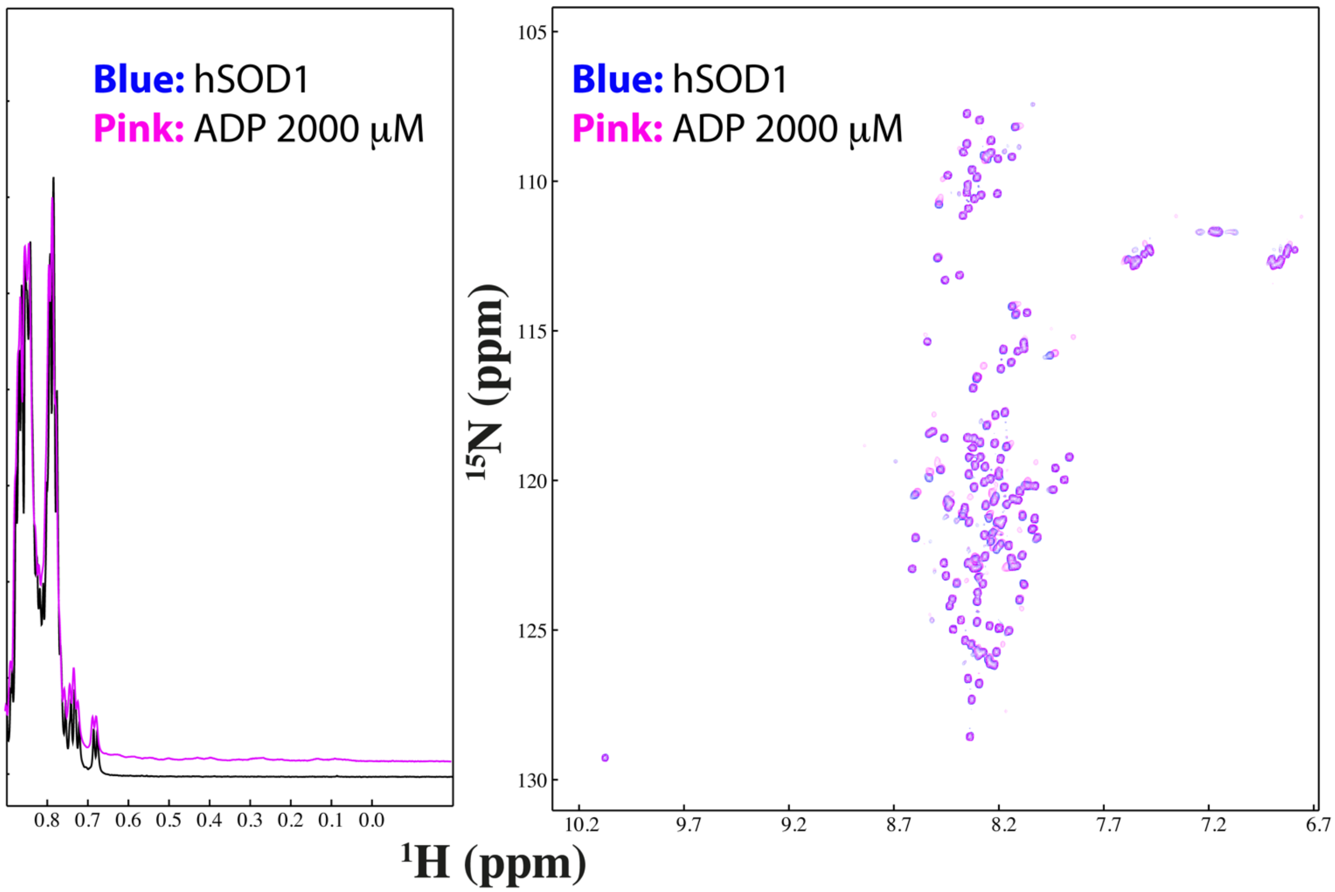

Supplementary Figure 20. ADP failed to induce folding of nascent hSOD1.
Up-field NMR 1D and HSQC spectra of ^{15}N-labeled hSOD1 at a concentration of 50 μM in the absence (blue) and in the presence of ADP at 1:40 (purple).
