# Supplementary material for: ATP induces folding of ALS-causing C71G-hPFN1 and nascent hSOD1
Source: Commun Chem. 2023 Sep 5;6:186. doi: 10.1038/s42004-023-00997-0 (PMC10480188; doi:10.1038/s42004-023-00997-0)
Supplement: Supplementary file 2 — Description of Additional Supplementary Files [file 42004_2023_997_MOESM2_ESM.pdf]

Description of Additional Supplementary Files

File name: Supplementary Data 1

Description: Raw data for Fig. 2c and 2c, Fig. 5c and 5d, Fig. 8a
